# ClinSV: clinical grade structural and copy number variant detection from whole genome sequencing data

**DOI:** 10.1186/s13073-021-00841-x

**Published:** 2021-02-25

**Authors:** Andre E. Minoche, Ben Lundie, Greg B. Peters, Thomas Ohnesorg, Mark Pinese, David M. Thomas, Andreas Zankl, Tony Roscioli, Nicole Schonrock, Sarah Kummerfeld, Leslie Burnett, Marcel E. Dinger, Mark J. Cowley

**Affiliations:** 1 Kinghorn Centre for Clinical Genomics, Garvan Institute of Medical Research, 370 Victoria Street, Darlinghurst, NSW Australia; 2 St Vincent’s Clinical School, UNSW, Sydney, NSW Australia; 3 Sydney Genome Diagnostics, The Children’s Hospital at Westmead, Hawkesbury Road & Hainsworth Street, Westmead, NSW Australia; 4 Genome.One, Darlinghurst, NSW Australia; 5 Children’s Cancer Institute, University of New South Wales, Randwick, Sydney, NSW Australia; 6 School of Women’s and Children’s Health, UNSW, Sydney, NSW Australia; 7 The Kinghorn Cancer Centre and Cancer Division, Garvan Institute of Medical Research, 370 Victoria Street, Darlinghurst, NSW Australia; 8 Department of Clinical Genetics, The Children’s Hospital at Westmead, Hawkesbury Road, Westmead, NSW Australia; 9 Sydney Medical School, The University of Sydney, Camperdown, NSW Australia; 10 NSW Health Pathology Randwick, Sydney, NSW Australia; 11 Centre for Clinical Genetics, Sydney Children’s Hospital, Randwick, NSW Australia; 12 Prince of Wales Clinical School, University of New South Wales, Sydney, NSW Australia; 13 Neuroscience Research Australia, University of New South Wales, Randwick, Sydney, NSW Australia; 14 School of Biotechnology and Biomolecular Sciences, UNSW, Sydney, NSW Australia

**Keywords:** Structural variation, Copy number variation, Whole genome sequencing, Microarray, Clinical genome, Rare disease

## Abstract

Whole genome sequencing (WGS) has the potential to outperform clinical microarrays for the detection of structural variants (SV) including copy number variants (CNVs), but has been challenged by high false positive rates. Here we present *ClinSV*, a WGS based SV integration, annotation, prioritization, and visualization framework, which identified 99.8% of simulated pathogenic ClinVar CNVs > 10 kb and 11/11 pathogenic variants from matched microarrays. The false positive rate was low (1.5–4.5%) and reproducibility high (95–99%). In clinical practice, *ClinSV* identified reportable variants in 22 of 485 patients (4.7%) of which 35–63% were not detectable by current clinical microarray designs. *ClinSV* is available at https://github.com/KCCG/ClinSV.

**Supplementary Information:**

The online version contains supplementary material available at 10.1186/s13073-021-00841-x.

## Background

Genomic structural variant(s) (SV(s)), including copy number variant(s) (CNV(s)), are an important source of genetic variation, and it is well established that large CNVs (typically > 100 kb) are an important cause of many inherited human genetic diseases [1–3]. Clinically accredited array comparative genome hybridization (aCGH) or single nucleotide polymorphism (SNP) microarrays (MA) are currently first-line clinical laboratory tests used to diagnose patients with many rare genetic diseases. Especially common among these conditions are intellectual disability [4] and autism [5]. Depending on their probe density, aCGH and SNP MA detect CNV down to a resolution of ~ 50 kb and are, except for loss of heterozygosity regions, unable to detect copy number-neutral SV events, such as inversions or balanced translocations. Short-read whole genome sequencing (WGS) has emerged as a comprehensive test for diagnosing rare inherited genetic disorders. A small number of laboratories have obtained clinical accreditation (e.g., CLIA/CAP [6], or ISO 15189 [7]) for the identification of single nucleotide variants (SNVs) and short insertion or deletion variants (indels) (< 50 bp) from WGS, where sensitivity and specificity often exceed 99% across most of the genome. Due to its broad and uniform sequencing coverage, WGS additionally has considerable potential to identify both small and large CNVs, with size ranging from smaller-than-single-exon, up to whole-chromosome aneuploidy, as well as copy-number-neutral SVs. Until recently, WGS analytical methods had imperfect sensitivity and specificity for detection of SVs, leading some groups to report that SV detection from WGS was not yet fit for use in clinical practice [8, 9]. There are now numerous recent reports using WGS to identify short CNV, affecting single genes, or even single exons as the cause of many monogenic disorders, suggesting considerable potential for short CNVs below the limit of detection of traditional MA to explain a proportion of previously undiagnosed patients [4, 10, 11]. The ability to find short, potentially pathogenic CNVs raises significant new interpretation challenges, as there are thousands of benign short CNVs in healthy individuals [12, 13].

**Fig. 1:**
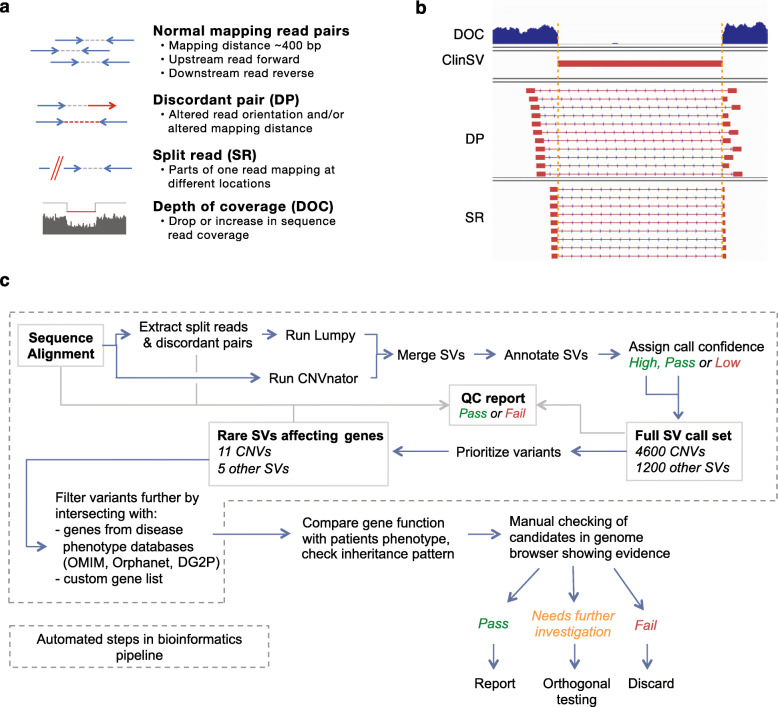
Evidence types used for CNV and SV identification, and the *ClinSV* workflow. **a** The different evidence types used for automated SV identification from short-read paired-end WGS data. **b** An example homozygous CNV deletion with clear breakpoints demarcated by a sharp drop in read coverage, and high numbers of supporting split reads (SR) and discordant read pairs (DP). **C:** The *ClinSV* workflow demonstrates the integration of two variant callers, variant annotation, quality classification, prioritization, and interpretation

For the detection of SVs and CNVs, short-read paired-end sequencing provides four main categories of evidence: changes in depth of coverage (DOC), reads with a gapped sequence alignment referred to as split reads (SR), reads-pairs with aberrant mapping orientation or distance referred to as discordant pairs (DP, Fig. 1a, b), and variant allelic fraction of heterozygous SNPs. Most CNV detection tools (e.g., CNVnator [14]) use only changes in DOC, while most popular SV detection tools (e.g., Lumpy [15], Delly2 [16] and Manta [17]) use evidence from SR and DP to identify SVs [16, 18–20]. Used alone, each of these methods gives an incomplete picture. DOC methods work well for large CNVs with confidently mapped reads but perform poorly for shorter CNVs, and for regions with poorly mapped reads, or extreme GC content. Recent improvements in DOC methods have demonstrated high-quality CNV calling from WGS down to 10 kb [20]. SR and DP can identify small CNVs and copy number-neutral SVs, often with base-pair precision, but perform poorly when supporting reads are ambiguously aligned, and suffer from reduced coverage at regions of extreme GC nucleotide composition. Previous studies have proposed to improve CNV and SV detection by integrating multiple variant callers [21–24]. These approaches often combine large numbers of individual CNV and SV callers to increase sensitivity, at the expense of specificity, complexity, and analytical cost, and do not address the challenges of variant visualization, annotation, and prioritization of rare variants.

Manual assessment of individual candidate SVs remains critical, with many groups using Integrated Genome Viewer (IGV) [25], or more specialized tools including svviz [26], SplitThreader [27], Ribbon [28], or SVPV [29]. However, most of these tools are limited to visualizing read evidence from single samples, ignoring additional lines of evidence to assess the quality, and clinical relevance of each candidate SV.

The variants that cause inherited genetic diseases are typically very rare in the general population. Deriving a database of SV frequencies however is challenging, as there is currently no consensus on the method or technology used to detect CNVs or SVs from genomic data. Furthermore, most databases of CNVs, such as the database of genomic variants (DGV) [30], include predominantly MA studies with varying resolutions making it difficult to determine whether partially overlapping CNVs represent the same event. To date, the most comprehensive resource of small and large CNVs derived from WGS is from the Genome Aggregation Database (gnomAD) [31], which was established using numerous CNV and SV callers. High-coverage whole genome sequencing data (30) has been recently generated from the openly consented participants from the 1000 genomes project [12], which will be an important open resource for CNV methods development.

Assessing the analytical performance of new SV detection methods is hampered by the lack of comprehensive and accurate reference call sets. While several validated SV datasets using the NA12878 reference cell line exist [15, 26, 32, 33], the concordance between three of them was only 33% [34]. This poor concordance is likely due to differences in the technology (e.g., MA, WGS, PCR) and analytical approaches used. More recently, long-read sequencing datasets provide more comprehensive gold standard call sets, which can now be used for calibration and benchmarking [33, 35].

To overcome the challenges associated with accurate CNV and SV detection for rare disease diagnosis, we have developed *ClinSV*, a SV integration, annotation, prioritization, and visualization framework. The focus of this method has been the detection of germline variants, as opposed to mosaic or somatic variants. We developed and optimized *ClinSV* through extensive in silico benchmarking experiments and comparisons to patients with matched MA from clinical laboratories. Through the analysis of 500 healthy elderly controls with 30–40x WGS, we compiled a population allele frequency database of CNV and SV, to assist with prioritizing rare, pathogenic CNV. *ClinSV* received ISO15189 clinical accreditation from National Association of Testing Authorities, Australia (NATA), in March 2018, and here, we report the use of *ClinSV* across 485 consecutive patients referred for WGS within a clinically accredited diagnostic genomics laboratory.

## Methods

### Reference materials, patient recruitment

DNA was obtained from the GIAB consortium from the NA12878 (GIAB1) and Ashkenazi trio (GIAB2) reference cell lines. DNA was provided for 11 patients from cohort A and 17 patients from cohort B whom were submitted for diagnostic testing at two Australian clinical laboratories for clinical microarray analysis. These samples were provided solely for the purpose of methods development and evaluating CNV performance, and do not have consent for the release of raw or processed genomic data. Four hundred eighty-five patients were referred to the Genome.One diagnostic laboratory for genomic testing (including CNV analysis) in a clinical setting and do not have consent for the release of raw or processed genomic data. WGS data from 500 healthy controls were provided by the Medical Genomics Reference Bank (MGRB) [36, 37].

### Genome sequencing and primary analysis

DNA samples were processed using standard Illumina TruSeq Nano HT v2.5 library preparation on Hamilton Star instruments and sequencing was performed on Illumina HiSeq X instruments at the Kinghorn Centre for Clinical Genomics at Garvan Institute of Medical Research, Sydney, Australia. Only samples passing sequencing QC were processed further, i.e., mean coverage > 30x (coverage calculation according to Illumina technical note), > 75% Q30 bases, and total yield > 100 Gb. Sequence reads were aligned to the human genome reference assembly GRCh37 decoy using BWA MEM (v0.7.12-r1039, settings -M). Aligned reads were sorted and duplicate-marked using Novosort (V1.03.01).

### *ClinSV* pre-processing and variant integration

Variants were processed individually or in batches of up to 15 samples. Discordant mapping read pairs (DP) and split reads (SR) were extracted from the sequence alignment file. To determine the upper and lower discordant mapping distance cutoffs, the mapping distance for all read pairs with normal mapping orientation in a sample region (1:1,000,001–5,000,000) was obtained by *ClinSV*. The read pairs were sorted by their mapping distance. Iterating through this list in descending order and summing the number of read-pairs, the upper cutoff was set to the mapping distance at an average coverage of 100 read pairs per Mb. The lower limit was obtained accordingly by iterating in ascending order. DPs were extracted from the whole BAM, using PE-SR-bam2bed.pl (part of *ClinSV*) and these cutoffs. SRs were extracted using the “extractSplitReads_BwaMem” script from Lumpy [15] and further processed with PE-SR-bam2bed.pl, discarding reads mapping to NC_007605 or hs37d5, reads having more than one clipped part, reads having overlapping alignment parts, or the clipped part having more than one alignment in the SA tag. Coverage was assessed using the get_coverages.py script, and an excluded region file was generated to exclude regions with > 300x coverage using the get_exclude_regions.py scripts from Lumpy. Lumpy v0.2.11 was run to detect genome-wide SV with the options -mw 3 -tt 0, using only the discordant and split reads. Resulting variant calls were annotated with depth of coverage (DOC, see the “Depth of coverage annotation” section below). When joint-calling multiple samples, in case a deletion or duplication had DOC support only for some samples and others samples only having DP/SR, then such variants were outputted as two separate variant calls, one CNV and one copy neutral variant (CNV = 0).

CNVnator v0.3 was run in parallel using cnvnator_wrapper.py v.0.0.1 [21] with a window size of 100 bases. CNVnator calls spanning masked regions (marked with Ns in FASTA) of the genome were split, excluding the masked part. CNVnator calls were annotated with DP and SR showing the expected mapping orientation [15] and being located within certain inner (towards the center of the CNV) and outer limits (flanking the CNV) of each breakpoint. The outer limit was set to 1% of CNV length but at least 1 kb and at most 5 kb. The inner limit was set to half the CNV length but at most 5 kb.

The genotype was assigned purely based on the DOC, since the percentage of DPs and SRs were found to be poor indicators for the actual genotype, due to repeats at breakpoints. A DOC normalized to the average coverage (abbreviated as DRA) between ≥ 0.2 and < 0.8 or > 1.2 and ≤ 1.75 were defined heterozygous (0/1) and a DRA < 0.2 or > 1.75 as homozygous (1/1). In case multiple samples were processed, CNVnator calls were first merged across samples requiring the same event type (gain or loss), and a reciprocal overlap of at least 70% or the non-overlapping parts of each call to be at most 200 bases. For merged calls, the average start and end coordinates, and call properties were calculated. CNVnator calls were then merged to Lumpy calls using the same merging criteria, preserving the more precise start and end coordinates of Lumpy’s SV calls that are based on SRs and DPs.

QC metrics from the input and output data of *ClinSV* are automatically collected for each sample and compared to the MGRB control cohort. The deviation from the control cohort’s average is reported as *z*-score. Samples passed QC if most input metrics were within an absolute *z*-score of 2.

To annotate SV with features, such as DGV variants or genes, features were stored in BED, VCF, or GFF format, indexed using tabix and queried using the Perl module tabix (https://github.com/samtools/tabix/tree/master/perl, v0.2.0).

### Depth of coverage annotation

For annotating SV calls with DOC, bam sequence alignment files were converted to BigWig [38] format and queried using the Perl module Bio-BigFile-1.07. The DOC of a variant was normalized by the DOC of its flanking region (DRF to DOC ratio flanking) or the genome average (DRA). DRF was calculated by dividing the DOC within the variant by the average of both flanking regions of the same length as the variant. The DOC for the DRF and DRA computation was determined if possible from genomic regions with an average read mapping quality of ≥ 50 (Phred scale; max 60 for BWA MEM), unless less than one third of the variant length fulfilled this criterion, in which case no mapping quality cut-off was applied.

The average genome read coverage was estimated from a 10-Mb window (20,000,001 to 30,000,000) in each autosome, which was chosen to not overlap any centromeric or telomeric regions. The autosomal read coverage was set to the median coverage of the 10-Mb region across all autosomes. For the Y chromosome, the coverage was estimated from the two large unique regions (6,641,419–10,079,253 and 13,800,704–23,668,908) and for X the entire chromosome was used, to be robust against large copy number changes. To further increase the robustness of the sex chromosome coverage estimates, their coverage was rounded in fractions of 0.5 of the autosomal coverage.

SV calls from Lumpy and CNVnator were classified as CNV (CNV = 1) if DRA or DRF was < 0.8 or > 1.2. The population coverage standard deviation was computed from DRA of 500 healthy control samples in adjacent intervals of 1 kb. Most of the human genome (85%) had a standard deviation of < 0.15 (Additional file 1: Fig. S1).

### Determining SV population variant allele frequencies

Five population allele frequency (PAF) estimates were calculated. Three were derived from a control cohort of 500 healthy Australian individuals as part of the Medical Genome Reference Bank [36, 37], one from gnomAD [31] and one from the 1000 Genomes Project [12] (PAF1KG, Additional file 1: Fig. S2).

One PAF derived from the MGRB cohort was based on the sum of DP and SR counts (referred to as PAFSU), another was based on the normalized read depth (referred to as PAFDRA), and the third was based on the actual Pass and High confidence variants (referred to as PAFV). MGRB variants were joint-called in batches of 15 samples. PAFSU was calculated in order to obtain an abundance estimate of variants with few DPs and SRs close to or below the detection threshold (Additional file 1: Fig. S3). PAFSU is computed from the sum of DP and SR supporting a variant (SU) and the corresponding sum of DP and SR in the control cohort (SUC) averaged by the number of control samples (NCS, PAFSU=SUC/SU/NCS). For example, if a variant has in total 10 supporting DPs and SRs, and if in the control cohort (*n* = 500), there are in total 200 matching DPs and SRs, then PAFSU is 4%. Control sample’s DPs and SRs had to be located within 1000 bases flanking each breakpoint and orientated corresponding to the SV type (DEL, DUP, INV). PAFDRA was calculated to obtain an abundance estimate for CNV in regions of segmental duplications lacking DPs and SRs often having imprecise boundaries or being fragmented (Additional file 1: Fig. S4). PAFDRA is obtained by counting the number of samples that show the same copy number change (gain or loss) over at least 90% of the CNV. To speed up the comparison process, the DRA of the control samples was summarized in adjacent intervals of 1000 bases. This summarized coverage was also used to compute the population coverage standard deviation. When determining concordant variants for assigning PAF1KG and PAFV, the variant type (DEL, DUP, INV) including the CNV state (CNV = 1 for gain or loss, CNV = 0 for copy number neutral calls) had to match. For copy number-neutral SV, each breakpoint position could deviate at most 1000 bases. For CNV, at least 20% of the reference variant length and 90% of *ClinSV* variant length had to match. For *ClinSV* variants smaller than 1000 bases, at least 70% of their length had to match.

### Variant prioritization

A variant was marked as common if any of the PAF values (PAFV, PAFSU, PAFDRA, or PAF1KG) were > 0.01; remaining variants were declared rare (RARE = 1). Variants affecting genes present in the provided gene list will contain the corresponding gene name in column CANDG. When jointly analyzing multiple individuals and providing pedigree information in ped format, column IA and IUA show how often a variant was present among affected and unaffected individuals, respectively.

### Additional SV annotation

*ClinSV* variants were also annotated with SV from DGV [30], using the same variant matching criteria as for PAF1KG. Additional SV annotation included the variant’s GC content and the sequence compression ratio (CR). The latter is a measure for the repetitiveness of a sequence and used to indicate tandem repeats. For this, the compressed sequence length (using perl Compress::Zlib) is divided by the actual sequence length. Accordingly, a tandem repeat region has a lower CR than a complex sequence. SVs were also annotated with previously published segmental duplications [39] obtained from genome.ucsc.edu. In cases where more than one segmental duplication overlapped with an SV, the best matching was selected, which was defined as the one with the highest sum of overlap length (in % of variant length) and sequence similarity (in %, sum of both). For annotating SV with genes they affected, ENSEMBL genes release 75 for GRCh37 [40] excluding pseudogenes was used. Gene to disease phenotype associations were obtained from OMIM, Orphanet, and DDG2P via ENSEMBL Biomart GRCh37 [40].

### Analytical performance—sensitivity using ClinVar

Pathogenic and likely pathogenic ClinVar variants between 50 bases and 1 Mb were obtained from the UCSC table browser in May 2020. Variants were inserted in silico into two GRCh37 reference sequences, one for the copy number losses including variants marked as deletions and one for gains including duplications. Variants were required to not overlap and respecting a 5-kb flanking region, prioritizing smaller over larger variants. Excluding 4 variants in masked regions of the genome build GRCh37, we obtained 2003 deletions and 467 duplications. Illumina paired reads were generated in silico from each reference using ART [41] to an average coverage of 15× depth and combined with an additional 15× depth of the unmodified reference excluding chromosome X and Y, resulting in two 22xy genomes heterozygous for autosomal variants and homozygous for variants on X and Y. Variants were detected with *ClinSV* using default settings. Variants were considered concordant if their breakpoints were within 1000 bases or if a reciprocal overlap of at least 80% of the variant length was observed, allowing multiple matching parts given all parts were HIGH or PASS.

### Analytical performance—sensitivity using GIAB

We assessed the sensitivity of *ClinSV* to detect CNVs using reference cell lines and gold standard variant callsets developed by the GIAB consortium for NA12878 (aka GIAB1) [33] and an Ashkenazi parent-child trio (aka GIAB2) [33]. We used independent long-read PacBio sequencing data [33] to filter out 12 CNV deletions > 500 bp from GIAB1 that lacked any support from both our short-reads and PacBio long-read sequencing data. The more recent GIAB2 was supplied without read depth information, so duplications were distinguished from insertions based on the uniqueness of the inserted sequence as flagged by GIAB. We sequenced NA12878 reference material nine times and the child from the Ashkenzi trio once. SVs were detected for *ClinSV*, Lumpy, CNVnator, Delly2 (v0.7.6) and Manta (v-1.1.1) with default parameters, from a single replicate of NA12878 processed as described above. Here we defined sensitivity as the fraction of true positives identified from the set of gold standard calls: TP/(TP + FN). Concordance criteria see sensitivity using ClinVar. *ClinSV* was originally developed using PCR-based TruSeq Nano data, but also evaluated on nine replicates of NA12878 PCR-free KAPA Hyper libraries. The observed sensitivity for deletions > 1 kb was neglibible (Nano 98.8% ± 0.4%, KAPA 98.7% ± 0.3%), and for deletions < 1 kb was 4.2% lower (Nano 85.5% ± 1.6%, KAPA 81.3% ± 0.7%). This may be due to slightly longer DNA fragment sizes in PCR-free libraries (data not shown).

### Analytical performance—false positive rate using PacBio

The same NA12878 Illumina short-read data was used as input to detect High and Pass variants for the integrative method *ClinSV*, and the variant callers Lumpy, CNVnator, Delly, and Manta. For each tool, 50 deletions and 50 duplications greater and smaller than 500 bases (200 variants per tool) were randomly selected and compared to the GIAB SV calls (deletions only), PacBio NA12878 SV calls, and PacBio read alignments. PacBio SV calls were obtained from the Genome in a Bottle (GIAB) resource web page (http://jimb.stanford.edu/giab-resources/ files last modified April 2015). These SV calls from GIAB were based on three different variant detection methods: PBHoney [42], a custom pipeline on assembled sequences (unpublished), and the Chaisson et al. methodology [43]. In addition, we called SVs with Sniffles v1.0.3, after realigning the PacBio reads using NGMLR v0.2.1 [44]. SV calls obtained through *ClinSV* or the GIAB gold standard [33] were considered valid, if they were present in at least one of the above PacBio variant call sets or if clearly visible in PacBio read alignment or read coverage profile, i.e., deletions and duplications smaller than PacBio read length were required to have SR support and those larger DOC support.

### Analytical performance—false positive rate using MLPA

Multiplex ligation-dependent probe amplification (MLPA) was used as orthogonal confirmation method to assess the false positive rate for *ClinSV* and to validate candidate CNV during the “application to clinical genetic testing” section below. MLPA assay setup was performed as per standard protocols which also included 4 control probes C1, C2 (binding to exons of EP300 and CREBBP), NR0B1 (binding chrX), and SRY (binding chrY) per batch. MLPA design was attempted for 2 probes for each variant as per standard protocols to allow redundancy in case of probe failure. CNVs not confirmed by MLPA were considered false positive calls.

### Analytical performance—reproducibility

Pass and High confidence *ClinSV* variants from nine NA12878 replicates were compared in an all versus all comparison. SV concordance was as for the GIAB1 sensitivity analysis.

### Analytical performance—comparison to microarrays

DNA samples of 11 patients from the molecular cytogenetics laboratory at South Eastern Area Laboratory, Sydney Australia (cohort A) were analyzed on the Agilent 8x60k ISCA v2 design array (031746). The design of this array consists of 59,059 distinct target probes and 3886 control probes. The array was semi-targeted, with 18,851 densely tiled probes located in cytogenetically relevant disease regions. Probes located in the “backbone” regions had a lower tiled density. Overall median probe spacing was 60 kb however this will be higher in regions of high probe density. Ten of 11 samples were run in a duplicate dye swap experiment design (one replicate sample labeled with Cyanine-3-dUTP, one replicate sample labeled with Cyanine-5-dUTP). The remaining sample was run in singlicate.

DNA samples of 17 patients from the molecular cytogenetics laboratory at the Children’s Hospital Westmead, Sydney Australia (cohort B) were analyzed on the Agilent 2 × 400 kb CGH array. This array comprises of 411,056 distinct biological probes and 5232 control probes with a median probe spacing of 5315 bp. Raw MA files were analyzed using Agilent CytoGenomics software v4.0.2.21 with the default calling algorithm (Default Analysis Method—CGH v2).

All MA calls were manually reviewed and classified as low, medium, or high confidence by highly experienced cytogeneticists (Additional file 1: Table S1). Low confidence calls were excluded from further analysis. Classification of confidence was based on minimum log2 deviation, minimum probes, frequency in the cohort B (high frequency indicative of potential MA artifact or copy number polymorphism), and the call being visually consistent with a copy number change (consistency of vertical scatter of probes, location within a chromosome). MA calls were lifted over from hg18 to the GRCh37 reference genome build. High and Pass confidence variants from *ClinSV* were compared to the MA calls, requiring at least 50% reciprocal overlap. Each discordant variant was visualized in a genome browser. CNVs with an overlap of 0–50% were reclassified as concordant imprecise.

### Flanking repeat characterization

We sequenced NA12878 by WGS (library FR05812606), ran *ClinSV*, and for each Pass CNV (*n* = 4634), the sequences surrounding each breakpoint were retrieved, including up-to 2 kb up and downstream. If the CNV was smaller than 4000 bases, the inner ends were trimmed to assure non-overlapping sequence pairs. For each CNV, the pair of breakpoint sequences were aligned to each other using NCBI blastn v2.3.0+ [45] (default settings). Repeats at the breakpoint obtained from the sequence alignment were required to cross each breakpoint by at least one base. To classify the repeats, Tandem Repeats finder (v4.0.9) [46] was applied to the upstream SV repeat using default settings. SV repeats for which no tandem repeat was identified were subsequently intersected with GRCh37 RepeatMasker annotation [47] using Bedtools (v2.25.0) [48].

### Application in clinical genetic testing

Patient blood samples were submitted for diagnostic testing to Genome.One, a clinically accredited laboratory in Sydney, Australia, from March 2018 to December 2019. DNA samples were extracted using standard protocols and subjected to WGS as described above, although some samples with suspected Autosomal Dominant Polycystic Kidney Disease used KAPA Hyper PCR-free library preparation kits. Following WGS and *ClinSV* analysis, CNVs for each patient were filtered by PAF1KG < 2% and PAFMGRB < 2% and restricted to high or pass confidence. The analysis was mostly restricted to disease-specific curated gene lists, or virtual gene lists consistent with the patient’s presenting phenotype; CNVs were filtered to only those overlapping these genes. Where whole genome analysis was requested, the analysis was limited to currently described disease-associated genes in OMIM. To allow for breakpoint uncertainty analysis was restricted to genic regions within +/− 20% of the total CNV size from the CNV breakpoints. This 20% value was a conservative “upper bound” based on the assessment of measurement uncertainty of CNV size performed during the clinical validation. The measurement uncertainty of the CNV size ranged from 0 to 20.9% in CNVs analyzed (data not shown). As would be expected, CNVs with split read and discordant pair evidence showed lower uncertainty with a median 0.11%, whereas CNVs with only DOC evidence showed a higher uncertainty with a median of 6.54%. The supporting data for CNVs in genes located outside of the breakpoints defined by the variant callers were critically reviewed to determine if there was evidence for the inclusion of the gene. While there were no such results in this study that were clinically reportable, in such an event the CNV would be confirmed by an orthogonal method, such as MLPA, prior to reporting. Evidence for intronic CNV calls was critically reviewed to determine the likelihood of a) including genic material b) disrupting splicing. For example, if split read evidence clearly defines the breakpoints as within the intron, there is a low likelihood that the CNV extends to the exon. Intronic CNVs were assessed for their proximity to acceptor and donor splice sites. Branch point motifs were not considered in the assessment of CNVs.

The CNV interpretation considered the overlap between the patient phenotype and that described for the disease associated with the deleted or duplicated gene, inheritance model of the disease, zygosity of the CNV, and any other SNVs or indels detected by standard pipelines. Classification of CNVs was based on a combination of the ACMG standards and guidelines for the interpretation of sequence variants [49] and the ACMG standards and guidelines for interpretation and reporting of postnatal constitutional copy number variants [50]. Variants were described using HGVS v15.11.

### Resolution of WGS CNV by clinical microarray

The major Australian accredited clinical diagnostic laboratories were surveyed to identify commonly used array designs for routine diagnosis of genetic diseases and the minimum number of probes required to call a CNV. Five array designs were used across seven laboratories (Additional file 1: Table S2). Array designs were downloaded from vendor websites and where required converted to bed file format. CNVs detected by WGS were converted to BED file format and bedtools intersect was used on each array design to extract the probes within the breakpoints of each CNV. Each CNV was deemed undetectable for an array design if it contained fewer than the minimum required probe number for a call within the breakpoints of the CNV.

## Results

### *ClinSV*

To identify clinically relevant CNVs and copy number-neutral SVs from Illumina short-read WGS data, we developed *ClinSV*, which is an SV integration, annotation, prioritization, and visualization framework, summarized in Fig. 1c, and described here.

#### Input

The input for *ClinSV* is a coordinate sorted duplicate-marked sequence alignment file, in BAM format. Indel realignment and base quality score recalibration are not necessary.

*Variant Identification: ClinSV* executes the variant callers Lumpy and CNVnator to produce sets of candidate variant calls, which are then annotated and integrated. Samples can be analyzed individually or jointly in batches of up to 15 samples. CNVs larger than 50 bp were detected using three evidence types, DP, SR, and changes to DOC (Fig. 1a), while copy number-neutral SVs were detected using just DP and SR. SRs generally allow detection of genomic breakpoints with base-pair resolution (Fig. 1b). *ClinSV* integrates CNV calls from CNVnator [14], which uses evidence only from DOC, and Lumpy [15], which uses evidence only from DP and SR. *ClinSV* then determines the level of SR and DP support for each candidate CNV from CNVnator and computes the change in DOC for each candidate CNV from Lumpy before merging overlapping calls (see methods). This supports the identification of higher confidence CNV calls with multiple lines of support.

#### Variant quality

The merged variant calls are assigned an automated confidence category (high, pass, low) based on the amount of supporting evidence and CNV size (Additional file 1: Table S3). Based on 30–40x WGS from Illumina HiSeq X, we defined the following criteria: High confidence CNVs are either large (> 100 kb), or > 10 kb with confidently mapped reads (i.e., average mapping quality > 55 across the entire variant). These criteria reduce false positives due to segmental duplications (Additional file 1: Fig. S5, S6). Pass CNVs are either > 10 kb or ≤ 10 kb with at least two supporting split or discordant read pairs. High confidence copy number-neutral SVs need at least 10 split or spanning read support, and at least one from each source, whereas Pass copy number-neutral SVs need at least 6 supporting reads. All other variants are flagged as Low confidence.

#### Quality attributes

Quality attributes of each candidate SV are reported, including the average mapping quality at breakpoints and across the SV, the average %GC content, the sequence complexity via its compression ratio (see methods), and overlapping segmental duplications [39]. Low mapping quality or extreme %GC indicates possible false positives due to mapping artifacts or GC-coverage bias. CNVs with small sequence compression ratios usually represent variants in highly identical repeats, which often have reduced DP and SR evidence from short-read sequencing (see below).

#### Annotation

SVs are annotated with variant allele frequencies from 500 healthy controls (see below), overlapping genes, as well as their known disorders from OMIM [51], and phenotypes as Human Phenotype Ontology (HPO) terms [52] from Orphanet [53].

#### Variant filtration

*ClinSV* identifies 4730 ± 190 CNVs (mean ± stdev), and 1100 ± 150 balanced SVs per germline 30–40x genome. While *ClinSV* does not systematically classify the copy number-neutral SVs into subtypes, the 1100 SV are mostly mobile element insertions (MEIs), ~ 50 inversions, and almost no translocations; these findings are consistent with previous genome-wide estimates from short-read sequencing data [15]. By restricting to rare, gene-affecting variants, *ClinSV* obtains on average 11 CNVs and 5 copy number-neutral SVs per individual. *ClinSV* also allows joint variant analysis of multiple samples, so if pedigree information is provided, variants that are associated with only affected individuals can be readily identified. If a set of candidate genes is supplied, this set is further reduced; for example, in ~ 100 familial dilated cardiomyopathy genes, on average only 0.5 rare SVs were identified per patient [10]. Finally, *ClinSV* annotates variants using OMIM and Orphanet terms which can be useful to prioritize variants in patients’ with challenging phenotypes.

#### *ClinSV* output files

*ClinSV* produces a text file of all annotated CNVs and SVs, and a smaller file with just the rare, gene-affecting CNVs and SVs (Additional file 2, Additional file 1: Table S4). To assess whether the input data and *ClinSV* results are within expectations, a comprehensive QC report is also generated, including metrics on the fragment length distribution, chimeric read counts, read coverage variability, genome wide DOC, number of variants stratified by caller, variant type, and properties including the frequency of gene-affecting and rare SVs (Additional file 3). Additionally, when run on the NA12878 cell line, a comprehensive analytical validation report summarizing sensitivity and reproducibility is produced. Metrics outside 2-standard deviations from the average value determined from 500 healthy control samples are flagged for review.

#### Visualization framework

**Fig. 2:**
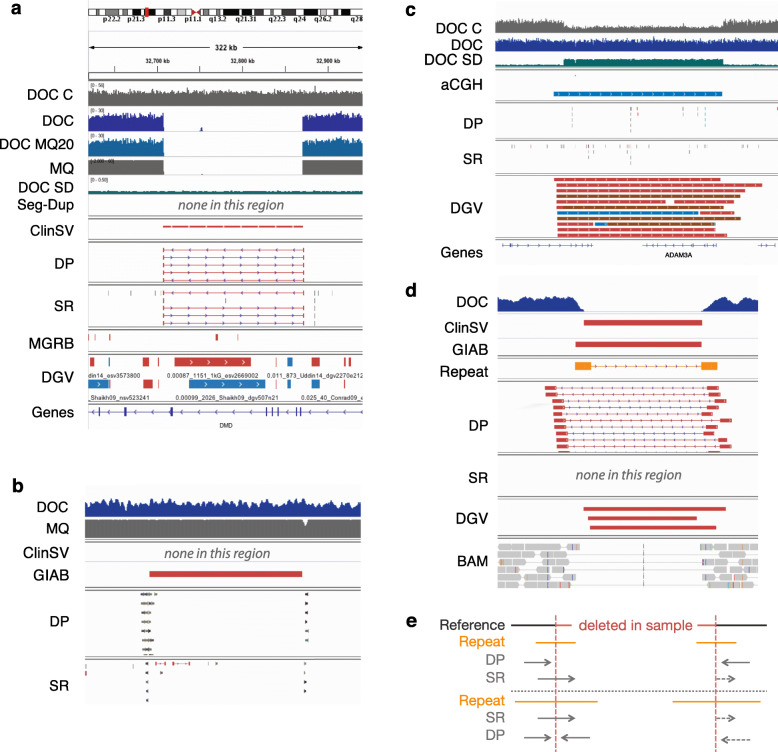
Properties and supporting evidence for accurate structural variant identification. **a** A pathogenic homozygous deletion in the Dystrophin gene. *ClinSV*’s default annotation tracks are: depth of coverage in an NA12878 control (DOC C), all reads in the sample (DOC), or reads with mapping quality ≥ 20 (DOC MQ20), the average mapping quality of aligned reads from the sample (MQ)**,** coverage standard deviation from 500 controls (DOC SD), segmental duplications [39] (Seg-Dup), annotated *ClinSV* calls (*ClinSV*), discordant pairs (DP), split reads (SR), *ClinSV* variant calls from 500 controls (MGRB), variants from the Database of Genomic Variants (DGV), and RefSeq genes (Genes). **b** A representative false positive CNV deletion identified by GIAB: the 5′ breakpoint is a mobile element insertion (MEI), the 3′ breakpoint is located in a repeat as suggested by the lower read mapping quality, and there is no intervening drop in coverage or mapping quality. **c** A representative false positive duplication called by aCGH not supported by WGS. The region shown is deleted in 44% of the samples in the 1000 Genome Project and coverage is frequently altered in controls (see DOC SD), but not in the actual sample (see DOC). **d** A homozygous deletion identified by *ClinSV* with reduced DOC, lots of DP support, but no SR support. The CNV is flanked by a pair of repeats. **e** A model demonstrating how CNV surrounded by repeats can reduce DP and SR support. For repeats greater then read length but smaller than paired-end fragment size only SR support is reduced (top, as in panel **d**), for repeats greater than the fragment size, both SR and DP support are reduced (bottom). The dashed arrows indicate the expected mapping location of DPs and SRs if the genomic region was not repetitive

Manually reviewing candidate variant calls remains a common practice in most research and clinical laboratories and is particularly important for SVs. *ClinSV* creates an IGV session file, which loads 11 tracks allowing the assessment of variant validity and potential clinical relevance (Fig. 2). We have provided recommended criteria to evaluate whether each candidate variant passes, needs further investigation, or is a likely false positive (Additional file 1: Table S5).

#### Orthogonal validation

Variants passing manual review were orthogonally confirmed using either MLPA or Sanger sequencing.

#### Scope

*ClinSV* identifies CNVs > 50 bp in length, and copy-number neutral SV if breakpoints are located in unique regions of the genome. *ClinSV* has been developed predominantly to support rare disease diagnosis workflows within the nuclear genome, but could identify SV in the mitochondrial genome, mosaic variants, and tumor genomes given enough supporting evidence, which will be evaluated more formally elsewhere.

#### Performance

Starting from a coordinate-sorted 30–40x depth BAM file, *ClinSV*’s runtime is 8 h on a single compute node (48 GB RAM, 16 CPUs, Additional file 1: Table S6). This fast performance is facilitated by rapid extraction and processing of SR and DP reads, and by running a parallelized version of CNVnator from SpeedSeq [21]. *ClinSV* can be run on any commercial or academic computing environment that supports Docker or Singularity, or instead by installing its dependencies.

### Allele frequencies from healthy individuals

To assist with the prioritization of rare SVs, we ran *ClinSV* on a cohort of 500 healthy individuals, sequenced with the same technology (i.e., 30–40x depth WGS from a single-lane of Illumina HiSeq X) and TruSeq Nano HT v2.5 library preparation chemistry. These healthy individuals were sequenced as part of the Medical Genome Reference Bank (MGRB [36, 37]). We identified 922,161 high-, 1,998,221 pass-, and 1,991,316 low-quality non-unique SVs across the cohort. We also evaluated the raw supporting read evidence, as highly repetitive parts of the genome may have highly variable or poor coverage, or have few split or spanning reads. We considered the raw evidence from SR, DP, and DOC, as well as final *ClinSV* variants, to derive three population allele frequency (PAF) measures from MGRB (see the “Methods” section). The underlying database of raw evidence data is included in the *ClinSV* distribution (see files SR.brkp.gz and PE.brkp.gz) and the derived PAF capture common complex variants or mapping artifacts that often do not result into variant calls. In addition, we annotate variants with population allele frequencies from gnomAD [31] and the 1000 genomes project [12]. Variants are labeled as rare if none of the computed PAFs were greater than 1%, reducing the number of candidate variants to inspect > 100-fold.

#### Analytical validation

We assessed the analytical performance, including sensitivity, false positive rate and reproducibility of *ClinSV*, by comparing to pathogenic ClinVar variants, Genome in a Bottle (GIAB) consortium gold standard reference materials [33, 35], long-read sequencing, MLPA and clinical MA (Table 1). Each technology and derived call-set had its own strengths and limitations in representing the different SV types, sizes, and pathogenic variants.

**Table 1:** Performance summary *ClinSV*. Summary of *ClinSV’s* performance by metric, variant type, and size. Input variants were pass and high confidence. Determining the false positive rate and false negatives, input variants were further visually reviewed prior the performance analysis

Metric	Reference data or technology	Preceding visual review	Value	Results table
Sensitivity	Simulated ClinVar			Table 2
	Deletions	No	99.7% > 10 kb 88.2% < 10 kb	
	Duplications	No	100.0% > 10 kb 63.4% < 10 kb	
	GIAB1			
	Deletions	No	97.1% > 10 kb 87.8% < 10 kb	Additional file 1: Table S7
	GIAB2			
	Deletions	No	97.0% > 10 kb 71.4% < 10 kb	Additional file 1: Table S8
	Duplications	No	100.0% > 10 kb 2.3% < 10 kb	Additional file 1: Table S9
	Microarray	No	100% pathogenic CNVs > 99% any CNVs	
False positive rate	PacBio data	Yes	1.5% 4.5% incl. inconclusive	Additional file 1: Table S10
	MLPA	Yes	0% 10% incl. Inconclusive	Additional file 1: Table S11
False negatives	MLPA	Yes	30%	Additional file 1: Table S13
Reproducibility	WGS	No	99.1% high CNVs 83.9% high and pass SVs	Table 3

#### CNV sensitivity

The sensitivity was assessed using 2470 pathogenic deletions and duplications replicated in-silico and two GIAB standards derived from cell lines of healthy individuals (NA12878, NA24385). The sensitivity was measured in an automated fashion using PASS and High confidence variants without manual inspection.

In order to assess performance of *ClinSV* on structural variants that are clinically relevant, we identified 2470 pathogenic and likely pathogenic CNVs from the ClinVar database and generated a simulated 30x-depth WGS dataset, where each variant was simulated as being heterozygous (see the “Methods” section). Among the CNVs larger than 10 kb, all duplications (281/281) and all but two deletions (674/676) were detected with *ClinSV* (99.8% sensitivity). Both missed deletions were partially identified, where one was interrupted by a segmental duplication and the other was split in two and lacked flanking SR and DP support. Smaller than 10 kb the sensitivity was 83.4–96.5% for the deletions and 41.1–85.7% for the duplications (Table 2).

**Table 2:** ClinVar pathogenic CNV sensitivity analysis. The sensitivity of *ClinSV* was assessed using simulated WGS data representing 2470 pathogenic or likely pathogenic CNVs

Size range	Deletions			Duplications		
	# Variants	Sensitivity	# Missed	# Variants	Sensitivity	# Missed
0–500	753	83.4%	125	95	41.1%	56
500–1 k	104	91.3%	9	9	77.8%	2
1 k–5 k	328	94.5%	18	47	89.4%	5
5 k–10 k	141	96.5%	5	35	85.7%	5
10 k–50 k	278	99.6%	1	79	100.0%	0
50 k–100 k	123	100.0%	0	28	100.0%	0
100 k–500 k	207	100.0%	0	110	100.0%	0
500 k–5 M	69	98.6%	1	64	100.0%	0
Total	2003	92.0%	159	467	85.4%	68

**Fig. 3:**
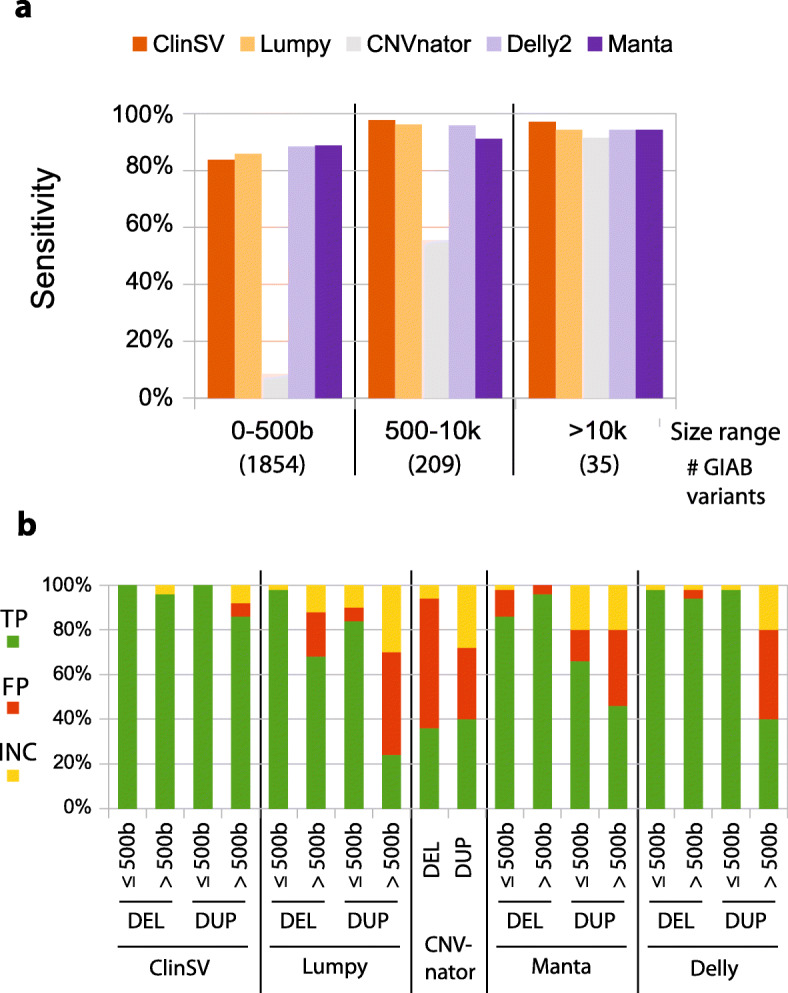
Analytical performance of *ClinSV*. We sequenced the NA12878 control cell line using WGS and compared the performance of a number of popular CNV or SV detection methods. **a** The sensitivity of different methods to identify 2664 CNV deletions from the Genome in a Bottle (GIAB1) gold standard call set over different size ranges. The number of deletions per size range is indicated along the bottom. **b** We selected deletions and duplications larger, or smaller than 500 bp (*n* = 50 each; total *n* = 200) from each of five different methods. CNVnator had few calls < 500 bp, so we combined all calls into one category. We assessed whether each call was a true positive (TP), false positive (FP) or inconclusive by comparing to long-read PacBio data

We also investigated *ClinSV's* sensitivity using two GIAB gold standard datasets (aka GIAB1, GIAB2, see the “Methods” section), where again the sensitivity also generally increased with larger size, reaching 97–100% for variants > 10 kb (Fig. 3a, Additional file 1: Table S7-S9). The overall sensitivity for detecting deletions was 88% for GIAB1 and 72% from GIAB2. Missed variants had little or no DP/SR evidence due to flanking repeats (Additional file 1: Fig. S7 and see below) or had conflicting DOC increase due to poor read alignment within the deleted repeat (Additional file 1: Fig. S8). GIAB1 was primarily based on Illumina short reads, whereas GIAB2 also included large amounts of long-read data, known to detect more than twice as many deletions and duplications in tandem repeats [43]. In comparison to the two popular SV callers Delly2 [16], Manta [17], *ClinSV* performed better for deletions > 500 bases but slightly worse for duplications (Fig. 3b, Additional file 1: Table S7–9). The overall sensitivity to detect presumably benign duplications < 10 kb, as represented by the GIAB2 standard was poor for all tested short-read based variant callers and *ClinSV* at 1.4–43.8% (Additional file 1: Table S9).

#### CNV false positive rate via PacBio

We evaluated the false positive rate (FPR) of CNVs identified by *ClinSV* and popular SV callers using the NA12878 cell line and CNV calls made using PacBio long-reads [44]. Deletions and duplications smaller or larger than 500 bp were randomly selected (50 each, total 200 per software) and categorized into three bins: present in control data (true positives), absent (false positives), and inconclusive. A variant was considered present in control if its boundaries coincided with a control variant visualized in IGV or was clearly present in visualized read alignment data (± 15% of length). A variant was considered inconclusive if the PacBio read data showed signs of a structural variant, but no clear DOC change or consistent gapped read alignments. The FPR is shown as a range, with the smaller number corresponding to the proportion of false positive calls to the sum of true and false positives, whereas the larger more conservative number counts inconclusive calls as false positives. The FPR was lowest for *ClinSV* (1.5–4.5%), followed by Manta (11.8–17.5%), Delly2 (17.9–26.5%), Lumpy (20.8–31.5%) and CNVnator (54.2–62.0%; Fig. 3b, Additional file 1: Table S10). 100% of the High confidence *ClinSV* deletions were true positives (*n* = 71), thus High confidence calls had a FPR of 0%. The visualization, annotation, and population allele frequency data, which support effective manual variant review, are critical elements of *ClinSV*, contributing to its lower FPR compared to the four callers.

#### CNV false positive rate via MLPA

The FPR of CNV detection from WGS using *ClinSV* was also assessed using custom MLPA which is a targeted assay for the detection of CNVs. From six patients, we selected 29 High and Pass CNV candidates that passed manual review, overlapped genes (+/− 200 bp), and were rare (PAF < 2%). Custom MLPA assay design was successful for 26 of these CNV candidates, all of which confirmed the presence of the CNV (FPR 0%, Additional file 1: Table S11). If we conservatively count the three CNV for which an MLPA assay could not be developed as false positives, then the FPR of *ClinSV* is 10.3%. Furthermore, we selected 10 gene-overlapping, rare CNV candidates that failed manual review (see visualization framework). Custom MLPA assay design was successful for three of the ten, with the assay confirming all three variants as true positives. These variants were duplications with a length between 36 and 93 kb, lacked DP/SR support, and overlapped annotated segmental duplications [39]. This highlights that such variants despite failing manual review—an important component of the *ClinSV* procedure—may still be true positives and that these variants, if located in relevant disease genes may warrant further investigation.

#### Reproducibility

We sequenced nine NA12878 replicates using three library batches, two different technicians, two sequencing runs, two sequencer sides, and two different lane positions (Additional file 1: Table S12). In an all-versus-all pairwise comparison totaling to 72 pairwise comparisons, on average 99.1% of high confidence CNVs and 98.7% of High confidence SVs were detected in at least one technical replicate (Table 3). When combining Pass and High confidence CNVs, the reproducibility was 85.0% and 83.9% for all SVs. A pairwise comparison of all replicates revealed similarly high concordance in all cases (Additional file 1: Fig. S9).

**Table 3:** Reproducibility of *ClinSV*. Nine replicates of NA12878 were sequenced using 30–40x WGS, and the consistency of CNV and SV calls identified by *ClinSV* was assessed

SVs/CNVs	Confidence	Average	Stdev	Min	Max
CNVs	High	99.1	0.3	98.5	99.7
CNVs	High and pass	85.0	1.9	81.3	88.8
All SVs	High	98.7	0.4	97.5	99.4
All SVs	High and pass	83.9	2.6	78.1	89.1

#### Concordance with patient microarrays

We compared CNVs detected by *ClinSV* from WGS to CNVs detected by clinically accredited MA. A total of 28 patients from two patient cohorts underwent WGS (Illumina HiSeq X 30–40x) and MA aCGH. One cohort of 11 samples consisted of patients with 11 pathogenic CNVs identified on Agilent 60 k CGH arrays (referred to as cohort A). The other cohort of 17 samples analyzed on Agilent 400 k CGH arrays (referred to as cohort B) had a total of 284 CNV calls, none of which was classified as pathogenic.

Of the pathogenic CNVs in cohort A, 100% (11/11) were identified using *ClinSV* (Table 4). Looking in more detail, CNVnator identified 9/11 CNVs, Lumpy identified 4/11 CNVs, and two sex chromosome aneuploidy events were identified only by *ClinSV*’s automated sex chromosome ploidy algorithm. Thus, even for the detection of large CNV within the size range of aCGH, it is critical to integrate evidence from DOC, DP, SR, and chromosome-wide coverage analysis (Additional file 3 section 3).

**Table 4:** Concordance of *ClinSV* with pathogenic variants from aCGH. Comparison of pathogenic CNVs identified by aCGH and WGS using *ClinSV* on matched DNA from the same patients. Eleven patients with known pathogenic variants were compared. Genomic coordinates are listed compared to the GRCh37 reference genome build. Sample A4 had a large CNV identified by aCGH, which was disrupted by a 7.3-kb diploid region in the middle

Sample	aCGH calls	WGS calls
A1	22q11.21 (18,894,835–21,505,417) × 1	del(22)(q11.21q11.21) chr22:g.18,873,701_21,466,500del
A2	22q11.21 (18,894,835–21,505,417) × 3	dup(22)(q11.21q11.21) chr22:g.19,025,201_21,502,400dup
A3	15q11.2q13.1 (23,656,936–28,559,402) × 1	del(15)(q11.2q13.1) chr15:g.23,679,001_28,659,900del
A4	15q11.2q13.3 (22,765,628–32,418,879) × 3–4	dup(15)(q11.2q13.2) chr15:g.22,727,651_30,670,600dup, dup(15)(q13.2q13.3) chr15:g.30,677,901_32,679,700dup
A5	(X) ×1	del(X)(p22.33q28) chrX:g.pter_cen_qterdel
A6	(X) ×2,(Y) ×1	dup(X)(p22.33q28) chrX:g:pter_cen_qterdup
A7	4p16.3 (1,832,643–1,994,922) ×1	del(4)(p16.3p16.3) chr4:g.1,830,325_1,996,237del
A8	16p11.2 (29,849,168–30,190,568) ×1	del(16)(p11.2p11.2) chr16:g.29,560,701_30,200,100del
A9	Xp22.33p22.31 (60,701–8,392,011) ×1, Yq11.221q12(16,188,682–59,335,913) × 1	der(X)t(X;Y)(p22.31;q11.221) g.[chrX:pter_8,434,700delinschrY:16,097,288_qterinv]
A10	Xp21.1 (32,714,410-32,868,014) × 0	del(X)(p21.1p21.1) chrX:g.32,707,584_32,871,077del
A11	15q11.2 (22,765,628-23,077,909) × 3	dup(15)(q11.2q11.2) chr15:g.22,727,651_23,377,700dup

We compared the total set of MA calls from either clinical laboratory to *ClinSV* variant calls from WGS and initially found a concordance rate of 62–75% (Additional file 1: Table S13). We manually inspected all of the *n* = 95 calls found only by aCGH. In cohort B, 64 of the 70 calls found only by aCGH were located in regions of common copy number variation, which often coincided with multi-allelic CNV [54], where the WGS data was clearly diploid (no DOC, SR, DP, in regions with good mapping quality). Since aCGH uses a pool of DNA from controls as a comparator, if a CNV is a common deletion in the general population, then a diploid patient may have an erroneous CNV amplification called (Fig. 2c), and vice versa (Additional file 1: Fig. S10). While these 64 events represent false positive calls in the MA data, in clinical practice, common copy number variants are usually filtered out, and not reported. In the cohort A, 4/25 discordant calls were in regions with common CNVs, 4/25 were not reproduced on the dye swap, 13/25 had less than four aCGH probes, 3/25 had poor logR intensity deviation from normal (2n) or probe intensities with a high standard deviation (Additional file 1: Table S13). There was one CNV call made by the 60 K aCGH array, of just 528 bp with more than 4 probes, for which we find no supporting evidence in the WGS data. Taken together, there was at most 1 *bona fide* extra call made by clinical MA that was not identified by *ClinSV*.

The limited probe density of MA affects the precision of the size of the resulting CNV calls [55]. *ClinSV* on WGS data was able to improve the precision of 38 of the 214 concordant calls from the cohort B, by a total of 960 kb (Additional file 1: Fig. S11). *ClinSV* also identified additional large CNVs that were not found in the MA data. In cohort B, there were on average 193 Pass and High confidence extra CNV per patient > 50 kb identified by *ClinSV*. Of these, 95% were in regions of frequent copy number variation in the population (population allele frequency > 5%), and thus potentially deliberately excluded from the MA probe design. The other 5% (average 7 per patient) were in repeat regions (average MQ < 34), which could thus be missed by MA due to challenges in designing probes for these regions, or false positives in the WGS data.

### Limitations of short-read based CNV detection

Despite all the improvements of short-read WGS based CNV detection over MA, short-reads have inherent limitations which can hamper CNV detection in some parts of the genome. We investigated why some CNV candidates lacked supporting SR and/or DP evidence (Fig. 2d, Additional file 1: Fig. S12, S13) and found that many had pairs of near-identical repeats on either side of the breakpoints. We hypothesized that in this scenario, paired-end read alignment algorithms would prefer to map the read pairs closer together than flanking the CNV (Fig. 2e). In NA12878, 13% (625/4634) of CNVs had flanking local repeat structures. The repeats ranged between 30 and 4000 bp and mostly consisted of tandem repeats (TR) (75%) followed by SINE (13%) and LINE (6%) elements (Additional file 1: Fig. S14, S15). The average number of SR, DP, and repeat length generally decreased with increasing sequence identity (Additional file 1: Fig. S15c).

### Application to clinical genetic testing

Collectively, these data demonstrated that *ClinSV* could identify CNVs from 30–40x depth Illumina WGS data with high reproducibility, sensitivity, and low false positive rates. Furthermore, *ClinSV* enables the identification of copy-number-neutral SVs and overlapping DEL-DUP events [10]. Ultimately these results were used to support the ISO15189 clinical accreditation of *ClinSV* for the analysis of CNVs from WGS data > 50 bp, in patients with suspected monogenic conditions.

**Fig. 4:**
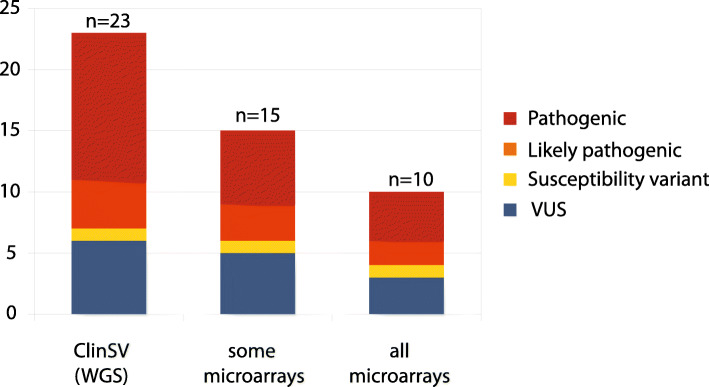
Clinically reported CNVs detected by *ClinSV* compared to callability by clinical microarrays. WGS with *ClinSV* analysis was applied in a diagnostically accredited laboratory to 485 patients with a diverse range of phenotypes and previous molecular testing. Twenty-three clinically reportable CNVs were identified and subsequently reported to the referring clinician. Only a subset of these would have been identified by all, or some of the microarray platforms available throughout clinical laboratories in Australia

Following ISO15189 clinical accreditation in March 2018 through the diagnostic genomics laboratory, Genome.One, *ClinSV* was applied to 485 patients with a diverse range of phenotypes including development delay, heart disease, hearing loss, neurological disease, and kidney disease. Within this cohort, a total of 23/485 patients (4.7%) had clinically reportable CNV, 100% of which were orthogonally confirmed, 21 using MLPA and two confirmed in alternative laboratories. MLPA or Sanger did not confirm one additional potential pathogenic CNV which had been classified as inconclusive following manual inspection. Of the reported CNVs, 52.2% were pathogenic, 17.4% likely pathogenic, and 30.4% were classified as variants of unknown significance (Fig. 4, Additional file 1: Table S14). The size range of the reported variants was between 500 bases to 1.5 Mb, with 35% being smaller than 10 k.

Four of the reported variants (ID 3, 5, 13, 22) were within genes associated with recessively inherited diseases, where the patients also had an additional rare SNV identified by WGS in the same gene, presumably in a compound heterozygous state: *ERCC5* (MIM133530), *STRC* (MIM606440), *SYNE1* (MIM608441), and *DST* (MIM113810). In these cases, the clinical report included a recommendation to perform family segregation analyses.

Of the clinically reportable variants 8/23 (35%) would not have been detected by any current commercial MA system commonly utilized in Australian clinical diagnostic laboratories and 13/23 (57%) would not have been detected by at least one commercial MA design. Of all pathogenic and likely pathogenic CNVs, 7/16 (44%) would not have been detected by any clinical MA, and 10/16 (63%) would not have been detected by at least one clinical MA design (Additional file 1: Table S14, S15).

The median size of CNV that would be unlikely to be detected by any MA was 3.8 kb (range 0.5–50 kb). The median size of CNVs that were detectable by at least one MA design was 424 kb (range 9.3 kb–1.5 Mb).

## Discussion

Here we have presented *ClinSV*, a platform that enables the accurate detection and interpretation of CNVs and SVs from short-read WGS, with a focus on identifying rare, pathogenic CNVs. To date, most approaches have treated CNV and SV identification as separate analytical challenges; however, as we have shown, there is substantial benefit in combining these approaches.

There are several features that contribute to *ClinSV*’s accuracy. First, it integrates complementary evidence from DOC, DP, and SR to obtain a comprehensive set of SVs and CNVs per sample. Secondly, the level of evidence supporting each candidate SV is combined with quality metrics to classify CNVs into high-, pass- and low-quality tranches. Excluding low-quality variants efficiently removed most false positives and kept the sensitivity high relative to other short-read callers. We posit that the poor performance of previous comparisons was due to low-quality CNVs affecting the overall quality. Thirdly, the filtration by numerous population allele frequency metrics calculated from a matched sequencing and software pipeline substantially reduced the number of candidate SVs from 5800 to an average of only 20 rare gene affecting variants per patient. Fourthly, the integrated visualization framework adds additional lines of supporting evidence from matched controls that facilitate manual inspection and further improves the performance of the method. And finally, the comprehensive quality control report is valuable to highlight when the quality of input WGS data is poor; WGS data with variability in sequencing coverage makes CNV identification very challenging and may be caused by degraded DNA or poor-quality sequencing libraries.

By comparing to extensive simulated data, benchmarking, and long-read sequencing data now available from the NA12878 and NA24385 cell lines, the analytical performance of *ClinSV* was strong. *ClinSV* detected 99.8% simulated pathogenic ClinVar CNVs > 10 kb and 85.1% < 10 kb. The increase in sensitivity for CNV > 10 kb is due to confident DOC based calls despite the absence of supporting DP and SR, which can occur due to repeats at variant breakpoints.

For presumably benign duplications and deletions > 10 kb the sensitivity was 97% (DEL GIAB1, DEL/DUP GIAB2) and < 10 kb ranging between 46 and 88% depending on the variant type and dataset analyzed. It was also shown that a copy number gain variant calls in regions of segmental duplication could be confirmed via MLPA, although failing manual review. We hypothesize that such variants are underrepresented in currently available validation call-sets, due to the difficulty to detect them. The majority of SVs in healthy individuals are short and in tandem repeats. In contrast, previously reported pathogenic variants have a higher proportion of variants > 10 kb (Table 2, Additional file 1: Table S7-S9). Long-reads are more sensitive for detecting short insertions and deletions in repeated regions of the genome [43]. Large amounts of such variants are included in the GIAB2 standard. *ClinSV* was developed using patient and control data derived from TruSeq Nano (PCR-based) library chemistry from Illumina HiSeq X sequencers. As sequencing chemistry, including PCR-free approaches and sequencing technology is evolving, this may limit the general applicability of this approach. However, we assessed the performance on PCR-free sequencing libraries, and sensitivity was comparable, especially for CNV > 1 kb. We recommend validating ClinSV*ClinSV* using data generated within your laboratory on the NA12878 cell line, and evaluating performance using the built-in quality control report that *ClinSV* provides.

*ClinSV*’s FPR was 1.5–4.5% overall, substantially outperforming other methods, and demonstrating good performance for CNV duplications, validated by long-read PacBio sequencing data. Variants passing manual inspection and for which an MLPA assay could be designed all validated. Finally, the reproducibility of high confidence CNV calls from *ClinSV* was 99.1%, and 85.0% for high and pass confidence variants. This reduction in reproducibility of Pass CNV (relative to High CNV) was usually due to variants with small number of supporting reads, close to the detection threshold, which occasionally fell below the automated detection cutoffs. This suggests that higher than 30–40x sequencing depth may improve reproducibility and increase the number of variant calls. The visualization of the region with a suspected variant may reveal additional supporting reads not sufficient to automatically call a variant.

One of the major challenges during the development of *ClinSV* was to obtain high confidence calls to benchmark our methods. Continued efforts by the community to share data [56], benchmark methods [57], and standardize variant call sets [33] are critical for methods development and will play an increasingly important part of translating genomics to the clinic. There are very few validated copy number-neutral SV, or sets of CNV duplications in NA12878 [33] or other healthy controls [12], so comprehensively assessing the performance of *ClinSV* and other software for these types of variants will require additional benchmarking resources to be developed. These may come from additional well-studied reference materials, studying very large cohorts with WGS, aggregating evidence from individual case reports, or benchmarking using cancer genome data. It will be an important goal to increase the number of validated copy number neutral structural variants in germline DNA to improve benchmarking of this class of genetic variant.

*ClinSV* was able to identify all pathogenic variants that were detected using MA by the two clinical laboratories participating in this study. The increased resolution of WGS compared to MA enables *ClinSV* to resolve large CNV with much higher precision, as well as identify thousands of CNV smaller than the resolution of MA, in addition to copy number-neutral SV. Absolute DOC measurements from WGS provide an advantage over relative quantification used in aCGH to accurately resolve the copy number level of regions frequently altered in the general population, often co-coinciding with multi-allelic CNV, which can include genes that are relevant to disease [58]. In support of this high clinical utility, *ClinSV* has been used in a number of research studies, which have identified short, pathogenic CNV affecting single exons [11], overlapping whole-gene deletion-duplications [10], and in adult patients with mitochondrial disease (Davis et al.*, in prep*).

By applying *ClinSV* to an unselected clinical cohort within a clinically accredited diagnostic laboratory we have demonstrated the increased clinical utility of CNV calling from WGS data over that of MA. Of particular interest are the 7/16 likely pathogenic and pathogenic CNVs that would not be readily detectable by MA. One could argue that reducing the minimum number of consecutive probes to make a diagnostic call by MA could help resolve some of these CNVs; however, this would still only recover at most an additional two CNVs (ID 4, 11) at the cost of significantly increasing the false positive detection rate of the assay. These small CNVs would be intractable to genome-wide MA techniques due to size and due to the heterogeneity of the disease within this cohort beyond the scope of targeted assays such as MLPA. An additional, underappreciated feature of expanding the variant types detectable by WGS is the consolidation of different variant types into a single assay. Cases 3, 5, 13, and 22 show a compound heterozygous state where one affected allele is a CNV and the other is a single nucleotide variant. Depending on the heterogeneity of the presenting disorder, the availability of previous test results, and the reporting policies of the laboratory it is plausible that these variants, if detected by different methods at different laboratories may be interpreted as single hits in a recessive disorder, and be overlooked and considered merely a carrier status rather than a diagnostic result when taken as a whole. Most demonstrative of this are the variants identified in *ERCC5* in case 5 where the presenting condition was complex and heterogeneous, the CNV was well below the resolution of MA and (to our knowledge) there are no commercially available MLPA kits to test for such a deletion. Without simultaneous SNV and CNV detection by WGS this patient is likely to have remained undiagnosed. While there have been limited studies showing that WGS performs similarly well compared to MA [20], to our knowledge this is the first publication showing clear clinical utility for genome wide CNV detection at higher resolution than MA.

The ability for *ClinSV* to identify short CNVs from WGS would represent an insurmountable interpretive challenge for clinical laboratories and researchers, without a way to differentiate the potentially pathogenic events from the thousands of polymorphic CNV present in all healthy individuals [12]. A critical advance of *ClinSV* is the use of matched population control data, with five complementary measures of PAF to prioritize rare CNV. The PAF measures derived from *ClinSV* calls in control (PAFV), gnomAD (PAFG), and the 1000-genomes project (PAF1KG) highlight the allele frequency of well-resolved CNV. Perhaps more importantly, the PAF developed from SR and DP raw data highlights genomic regions with ambiguous short-read-mapping, which are depleted from final CNV and SV calls, making this a powerful way to filter variants and common artifacts. Large CNVs affecting multiple genes can be interpreted as they currently are by cytogenetics and research laboratories, whereas smaller candidate CNVs should be assessed in the context of which genes are affected, the patient’s phenotype and family history, known gene-phenotype correlations, and sensitivity to gene dosage and haploinsufficiency. While recent recommendations [59, 60] will help to standardize this interpretation, it should be noted that the clinical interpretations in the issued pathology reports were performed prior to the publication of these guidelines and were instead based on a combination of previous guidelines [49, 50]. When combined with segregation analysis using additional family members, there are typically fewer than 10 variants to inspect.

We have also shown that short-reads are limited in their ability to resolve a subset of SV with repeated sequences surrounding each breakpoint, particularly if no DOC evidence exists (as for copy number-neutral SV) or if the DOC is not sufficient on its own (as for CNV < 10 kb). The most frequent repeat class found at breakpoints were tandem repeats and we hypothesize that these repeats may act as a catalyst to form SVs similarly as has been suggested for segmental duplications [61]. Long-read sequencing technologies including Oxford Nanopore and PacBio offer improved performance over repetitive elements, and aberrant GC%, and they can identify thousands of SV missed by short-read sequencing [43]. Long-read sequencing also enables complementary ways of detecting SV by using de novo assembly approaches, compared to the reference genome [62]. Optical mapping is able to identify even larger SV often missed by current long-read sequencing technology [63]. Strand-seq is another very promising technology allowing resolving haplotypes and detection of structural variation [64]. It is able to outperform long reads, short reads, and optical mapping for uncovering large inversions including inversions flanked by repeats, which are particularly challenging groups of structural variants [65]. Today, however, for most clinical and research laboratories seeking a single test that can accurately identify all SNVs, indels, and most CNVs and SVs from a patient’s genome, long-read sequencing remains cost-prohibitive at the sequencing depths required to also obtain robust SNV and indel performance. Supplementing high-quality short-read sequencing with limited amounts of long-read sequencing for CNV detection or using strand-seq may become a promising approach in the clinic.

## Conclusion

*ClinSV* can accurately identify pathogenic CNVs and copy number-neutral SVs from short-read WGS data in the size range from 50 bp up to whole-chromosome aneuploidy, at a resolution far higher than existing clinical microarrays. Through its integration, filtration, annotation, and visualization framework, it accurately identifies high-quality CNVs and SVs from patient clinical genome data and presents the data in a clinician- and researcher-friendly output. We have demonstrated that *ClinSV* combined with 30–40x short-read WGS provides increased clinical utility over microarray, resolving an additional 4.7% of cases over the usual standard combination of WES/WGS and microarray. By combining WGS with clinical-grade methods for SNV, indel, CNV, and SV detection, it represents a comprehensive, single-test capable of diagnosing a larger portion of genetic disorders than even a combination of previous best methods.

## Supplementary Information

**Additional file 1.** Supplemental Tables and Figures. Collection of supplemental Tables and Figures.

**Additional file 2.** Sample output rare gene affecting variants NA12878. ClinSV*ClinSV*’s output of rare gene affecting variants detected in control sample NA12878.

**Additional file 3.** Sample automated QC report NA12878. *ClinSV*’s automated QC report for control sample NA12878.

## Data Availability

The *ClinSV* software, license, installation and usage instructions, its supporting resource files, as well as Singularity and Docker images are available on GitHub [66]. The software is free for research and education purposes. For commercial or diagnostic usage inquiries, please contact the corresponding authors. The installation requirements are listed on the GitHub link above. The software is implemented in Perl and has been tested on Linux CentOS 8. MGRB SV evidence reads (SR and DP) and condensed variant calls needed for *ClinSV* variant annotation are available as described in the ClinSV*ClinSV* software distribution. Raw MGRB data is available upon application to the MGRB Data Access Committee, via https://sgc.garvan.org.au/terms/mgrb. The list of the 500 patients used in this study can be found at the ClinSV*ClinSV* software distribution page. In the validation study, patient samples were obtained through clinically accredited diagnostic laboratories and data used here for the purposes of methods development and validation was in accordance with permitted use of diagnostic samples for quality assurance and method improvement in such laboratories; these samples do not have consent for public release of matched microarray or WGS data.

## Abbreviations

aCGH: Comparative genomic hybridization array
CGH: Comparative genomic hybridization
CNV: Copy number variant(s)/variation(s)
DOC: Depth of coverage
DP: Discordant pair(s)
GIAB: Genome in a bottle
indel: Short insertion or deletion variant/variation
MA: Microarray
MLPA: Multiplex ligation-dependent probe amplification
MQ: Mapping quality
NATA: National Association of Testing Authorities, Australia
PAF: Population allele frequency
QC: Quality control
SNP: Single nucleotide polymorphism
SNV: Single nucleotide variant(s)/variation(s)
SR: Split read(s)
SV: Structural variant(s)/variation(s)
WGS: Whole genome sequencing
