# Supplementary material for: ClinSV: clinical grade structural and copy number variant detection from whole genome sequencing data
Source: Genome Med. 2021 Feb 25;13:32. doi: 10.1186/s13073-021-00841-x (PMC7908648; doi:10.1186/s13073-021-00841-x)
Supplement: Supplementary file 1 — Additional file 1. Supplemental Tables and Figures. Collection of supplemental Tables and Figures. [file 13073_2021_841_MOESM1_ESM.docx]

**Supplemental Tables**

**Table S1 aCGH call confidence criteria to assess the quality of a CNV**

| Confidence | Log2 deviation | Probes | Visual |
| --- | --- | --- | --- |
| High | ≥+0.36  ≤-0.6 | ≥5 | Consistent deviation with high probe density. Not in region of segmental duplication. Reproducible (If duplicate arrays available) |
| Medium | <+0.36  >0.6 | ≥3 | Some scattered deviation of probe. Visible on duplicate array (If duplicate arrays available) |
| Low | <+0.36  >0.6 | ≥3 | Scattering of probe density. Located within segmental duplication (or near centromere). Low probe density. Not present on duplicate array (If duplicate arrays available). |

**Table S2 Minimum number of probes per clinical microarray**

Major Australian diagnostic laboratories around the country were surveyed to identify commonly used array designs and minimum numbers of probes used for routine diagnosis of genetic diseases.

| Array Design | Minimum Probes |
| --- | --- |
| Illumina CytoSNP-12 | 15 |
| Illumina CytoSNP-850k | 15 |
| Illumina Infinium GSA v2 | 15 |
| Agilent SurePrint G3 ISCA v3 CGH+SNP 4x180k | 5 |
| Affymetrix CytoScan 750k | 25 |

**Table S3 Structural variant (SV) and copy number variant (CNV) call confidence criteria used by *ClinSV***

| **Value** | **Type** | **Criteria** |
| --- | --- | --- |
| High | CNVs | Criteria 1: DOC deviation > 20% and length > 100kb; or  Criteria 2: DOC deviation > 20% and length >10kb and Average MQ>55).  No SR or DP evidence required for criteria 1 or 2. |
|  | Copy-number neutral SVs | number of DP+SR > 10, DP > 0 and SR > 0 |
| Pass | CNVs | Criteria 1: DOC deviation >20% and a length >10kb; or  Criteria 2: DOC deviation >20% and the number of (DP+SR) >= 2 |
|  | Copy-number neutral SVs | number of (DP+SR) >= 6 |

**Table S4 The names and descriptions of the columns presented in the *ClinSV* result files**

| **Column name** | **Description** |
| --- | --- |
| family ^1, 2^ | Family ID from ped file (only if ped file was present for analysis) |
| pedInfo ^1, 2^ | Patient initials |
| affected ^1, 2^ | 2=affected, 1= unaffected (from ped file) |
| IA^1,2^ | Number of times a variant was detected **i**n **a**ffected individuals |
| IUA^1,2^ | Number of times a variant detected **i**n **u**n**a**ffected individuals |
| SAMPLE ^2^ | Internal sample ID |
| ID ^2^ | Variant ID |
| FT ^2^ | Automated call confidence **f**il**t**er column. Values HIGH, PASS, LOW |
| RARE | Is variant **rare**? (1=yes, 0=no) Rare means that PAFV, PAFSU, PAFDRA and PAF1KG are ≤ 1% |
| SU ^2^ | **Su**m of discordant pairs and split reads supporting the variant |
| PAFSU | **P**opulation variant **a**llele **f**requency estimated from **su**m of discordant pairs (DP) and split reads (SR) in control cohort. Control samples consist of 500 healthy elderly individuals from the Medical Genome Reference Bank (MGRB). Formula  (DP+SR control) / (DP+SR in sample) / (number of control samples). |
| PE | The number of supporting discordant **p**aired-**e**nd reads. This is what we refer to in the paper as DP: the number of **d**iscordant **p**airs. The VCF field ‘DP’ is reserved to report the read depth of a variant, so here we use the name PE, which was introduced by Lumpy. |
| SR | Number of supporting **s**plit **r**eads |
| DRF ^2^ | Read **d**epth **r**atio of variant vs **f**lanking regions |
| DRA ^2^ | Read **d**epth **r**atio of variant vs the **a**verage genome wide coverage  Copy number = DRA x 2 |
| PAFDRA | **P**opulation variant **a**llele **f**requency estimated from normalized **DRA** in control samples (MGRB cohort) |
| PCSD | **P**opulation **c**overage **s**tandard **d**eviation of control cohort |
| GT | **G**eno**t**ype estimation |
| MQBP | Average read **m**apping **q**uality of reads supporting both **b**reak**p**oints |
| CNV ^2^ | Is the structural variant a **CNV**? 1 = yes, 0 = no. Yes if DRA or DRF <0.8 or >1.2 |
| IGV ^2^ | Link to load **IGV** session file.  IGV needs to be open for this to work, and only needs to be run once per session. |
| GOTO ^2^ | Link to **go to** the region containing the variant in IGV |
| LOCATION ^2^ | Genomic **location** (chr:start-end) |
| SVTYPE ^2^ | **S**tructural **v**ariant **type**: Deletion, duplication, inversion or break ends (BND). BNDs represent a pair of breakpoints and can represent a translocation. |
| SVLEN ^2^ | **Len**gth of **s**tructural **v**ariant, in base pairs |
| TOOL ^2^ | Variant detection **tool:** Lumpy and/or CNVnator |
| PAFV ^2^ | **P**opulation variant **a**llele **f**requency from **v**ariants in control. At the time of publication, this is 500 healthy individuals from the MGRB cohort (https://sgc.garvan.org.au/initiatives/mgrb). |
| PAFG ^2^ | **P**opulation variant **a**llele **f**requency from **v**ariants in gnomAD |
| PAF1KG | **P**opulation variant **a**llele **f**requency in **1000** **g**enome project |
| GC | **GC** content of the variant |
| CR | Size **r**atio of **c**ompressed vs. uncompressed reference sequence of the variant. Low complexity sequences have smaller compression ratios. |
| MQ | **A**verage read **m**apping **q**uality of the variant |
|  |  |
| SEGD | Overlapping **seg**mental **d**uplications published by Bailey JA et al. 2002. For best match: % variant coverage \| % seg-dup coverage \| identity \| for all matching seg-dup’s: count \| merged % variant coverage |
| NUMG ^2^ | **Num**ber of **g**enes affected by the variant |
| GENES ^2^ | ENSEMBL genes affected by the variant |
| GFEAT | **G**ene **feat**ure affected by structural variant. If multiple features or genes affected, one feature is reported in order of importance (start_codon > stop_codon > CDS > UTR > intron) |
| HPO | **HPO** numbers of affected genes. HPO’s of genes are separated by the “\|” symbol and appear in the same order as the gene names in the GENES column. Multiple HPO’s per gene are separated by colon. |
| PHEN ^2^ | Known **phen**otypes for any genes affected by the variant, obtained from OMIM, DDG2P or Orphanet. If annotation from more than one source is available for a gene, only first source in above order is shown to reduce redundancy of terms. |
| CANDG ^2^ | Gene names that are also in the **cand**idate **g**ene list (if provided) |

^1^ If pedigree file was provided

^2^ the minimum set of annotation columns provided in the light version of this file

**Table S5 Recommended manual validation criteria for assessing the validity of a SV call**

| Value | SV type | Required  True | Required  False | Optional  True |
| --- | --- | --- | --- | --- |
| Pass | CNV lacking SR and DP | cCNV | cCC | cMQ |
|  | CNV with SR and DP | cDP/SR | cCC | cMQ, cCNV |
|  | Copy-number neutral | cMQ and cDP/SR | cCC |  |
| Needs further investigation | Any | cCC |  |  |
|  | CNV lacking SR and DP | cCNV | cDP/SR |  |
|  | Copy-number neutral |  | cMQ or DP/SR |  |
| False call | CNV lacking SR and DP |  | cCNV |  |
|  | CNV with SR and DP |  | cCNV and DP/SR |  |
|  | Copy-number neutral |  | cMQ and cDP/SR |  |

| cCNV: | CNV call has a distinct change in DOC, i.e. breakpoints are marked by a distinct change in DOC or DOC change of entire CNV stands out relative to flanking region. |
| --- | --- |
| cDP/SR: | Supporting DP and SR have diverse mapping starts and mapping ends not reaching into the breakpoint. SR supporting reads are consistently soft-clipped at breakpoint |
| cMQ: | Read evidence is in a region with high MQ (>40), i. e. DP, SR or reads within CNV |
| cCC: | Conflicting additional SR and DP in close vicinity of breakpoint or complex variant |

**Table S6 *ClinSV* runtime performance**

The *ClinSV* average runtime on a single compute node. The server hardware used had Intel Xeon Sandy Bridge 2.6 GHz architecture with 16-cores, and 48 GB RAM. The amount of RAM needed by Lumpy increases by number of samples and amount of input DP and SR. Calculating with 6GB per sample is generally sufficient.

| Step | CPU | MEM [GB] | Time [min.] |
| --- | --- | --- | --- |
| Create wig | 16 | 2 | 125 |
| Create MQ 0 coverage bigwig | 1 | 34 | 38 |
| Create MQ 20 coverage bigwig | 1 | 34 | 38 |
| Create MQ bigwig | 1 | 34 | 29 |
| CNVnator | 16 | 11 | 30 |
| Lumpy pre-processing | 2 | 4 | 188 |
| Lumpy | 1 | 1 | 8 |
| Lumpy post-processing | 12 | 1 | 4 |
| SV merging and annotation | 1 | 2 | 12 |
| Max/total | 16 | 34 | 472 |

**Table S7 Sensitivity for the detection of GIAB deletions**

The sensitivity of various CNV and SV detection methods to detect 2,664 CNV deletions from the GIAB1 gold standard [33], over seven size ranges.

| Size region | # Variants | ClinSV | Lumpy | CNVnator | Manta | Delly2 |
| --- | --- | --- | --- | --- | --- | --- |
| 0-500 | 1854 | 83.7% | 85.9% | 8.5% | **88.7%** | 88.4% |
| 500-1k | 209 | **96.2%** | 94.7% | 32.1% | 84.7% | 92.8% |
| 1k-10k | 566 | **98.1%** | 96.6% | 61.0% | 93.5% | 96.8% |
| >10kb | 35 | **97.1%** | 94.3% | 88.6% | 94.3% | 94.3% |

**Table S8 Sensitivity for the detection of GIAB2 deletions**

The sensitivity of various CNV and SV detection methods to detect 3,124 deletions from the GIAB2 gold standard [35], over seven size ranges. The missing 50k–100k deletion was called by CNVnator and present in ClinSV but split in two parts due to a 4.5kb gap spanning a repeat.

| Size region | # Variants | ClinSV | Lumpy | CNVnator | Manta | Delly2 |
| --- | --- | --- | --- | --- | --- | --- |
| 0-500 | 2387 | 66.3% | 68.5% | 6.9% | 75.7% | **76.4%** |
| 500-1k | 198 | **82.3%** | 80.8% | 33.3% | 69.7% | 81.8% |
| 1k-10k | 506 | **91.5%** | 89.7% | 62.1% | 83.6% | 89.7% |
| >10kb | 33 | **97.0%** | 93.9% | 87.9% | 90.9% | 93.9% |

**Table S9 Sensitivity for the detection of GIAB2 duplications**

The sensitivity of various CNV and SV detection methods to detect 1,760 duplications from the GIAB2 gold standard [35], over seven size ranges.

| Size region | # Variants | ClinSV | Lumpy | CNVnator | Manta | Delly2 |
| --- | --- | --- | --- | --- | --- | --- |
| 0-500 | 1681 | 1.4% | 1.4% | 0.0% | **5.8%** | 1.4% |
| 500-1k | 61 | 18.0% | 18.0% | 0.0% | 9.8% | **24.6%** |
| 1k-10k | 16 | 37.5% | 37.5% | 31.3% | 37.5% | **43.8%** |
| >10kb | 2 | 100.0% | 100.0% | 50.0% | 100.0% | 100.0% |

**Table S10 False positive rate of *ClinSV* assessed using PacBio**

The False positive rate of *ClinSV* was assessed using PacBio long-read sequencing data. The NA12878 cell line was sequenced, and analyzed using *ClinSV*, Lumpy, CNVnator, Delly and Manta. 200 randomly selected variants from each caller were selected, then manually inspected and compared to PacBio sequencing data from the same cell line. Calls were classified into true positives (TP), false positives (FP), and inconclusive (INC) on the basis of support from PacBio reads. For CNVnator, the majority of calls were greater than 500 bases, thus the distinction between smaller and greater than 500 bases were not made. The false positive rate (FPR) is listed as a range, whether inclusive calls are included as false positives or not.

| Software | Del/Dup | Size  [bp] | Confirmed (TP) | Absent (FP) | Inconclusive (INC) | FPR (%) |
| --- | --- | --- | --- | --- | --- | --- |
| ClinSV | DEL | <500 | 50 | 0 | 0 | 0.0 - 0.0 |
|  |  | ≥ 500 | 48 | 0 | 2 | 0.0 - 4.0 |
|  | DUP | <500 | 50 | 0 | 0 | 0.0 - 0.0 |
|  |  | ≥ 500 | 43 | 3 | 4 | 6.5 - 14.0 |
|  | Total |  | 95.5 % | 1.5 % | 3.0 % | 1.5 - 4.5 |
| Lumpy | DEL | <500 | 49 | 0 | 1 | 0.0 - 2.0 |
|  |  | ≥ 500 | 34 | 10 | 6 | 22.7 - 32.0 |
|  | DUP | <500 | 42 | 3 | 5 | 6.7 - 16.0 |
|  |  | ≥ 500 | 12 | 23 | 15 | 65.7 - 76.0 |
|  | Total |  | 68.5 % | 18.0 % | 13.5 % | 20.8 - 31.5 |
| CNVnator | DEL | ≥ 200 | 18 | 29 | 3 | 61.7 - 64.0 |
|  | DUP | ≥ 200 | 20 | 16 | 14 | 44.4 - 60.0 |
|  | Total |  | 38.0 % | 45.0 % | 17.0 % | 54.2 - 62.0 |
| Delly2 | DEL | <500 | 43 | 6 | 1 | 12.2 - 14.0 |
|  |  | ≥ 500 | 48 | 2 | 0 | 4.0 - 4.0 |
|  | DUP | <500 | 33 | 7 | 10 | 17.5 - 34.0 |
|  |  | ≥ 500 | 23 | 17 | 10 | 42.5 - 54.0 |
|  | Total |  | 73.5 % | 16.0 % | 10.5 % | 17.9 - 26.5 |
| Manta | DEL | <500 | 49 | 0 | 1 | 0.0 - 2.0 |
|  |  | ≥ 500 | 47 | 2 | 1 | 4.1 - 6.0 |
|  | DUP | <500 | 49 | 0 | 1 | 0.0 - 2.0 |
|  |  | ≥ 500 | 20 | 20 | 10 | 50.0 - 60.0 |
|  | Total |  | 82.5 % | 11.0 % | 6.5 % | 11.8 - 17.5 |

**Table S11 False positive rate of *ClinSV* assessed using MLPA**

False positives were determined from rare, gene affecting CNVs using MLPA. If counting *ClinSV* variants passing visual inspection and successful MLPA probe design the false positive rate was 0% (0/26) if including variants failing MLPA probe design 10% (3/29).

| Sample | Number of rare, genic variants | Number passing visual inspection | Number passing visual inspection with MLPA design | Number failed visual inspection with MLPA design | Number CNVs confirmed via MLPA of all with MLPA design |
| --- | --- | --- | --- | --- | --- |
| S1 | 7 | 5/7 | 5/5 | 0/2 | 5/5 |
| S2 | 9 | 5/9 | 4/5 | 1/4 | 5/5 |
| S3 | 12 | 10/12 | 10/10 | 1/2 | 11/11 |
| S4 | 4 | 4/4 | 3/4 | 0/0 | 3/3 |
| S5 | 7 | 5/7 | 4/5 | 1/2 | 5/5 |
| Sum | 39 | 29/39 | 26/29 | 3/10 | 29/29 |

**Table S12 Sample properties for testing reproducibility on NA12878**

Nine replicates of NA12878 were sequenced using three library batches, two different laboratory technicians, two sequencing runs, two sequencer sides, and two different lane positions.

| Repl. ID | Flowcell and Lane | Library Batch | Library | Operator | Seq. Run | Seq. Side | Lane Position |
| --- | --- | --- | --- | --- | --- | --- | --- |
| S1 | H7LH3CCXX_5 | 150711 | FR05812598 | X | 150901 | A | Internal |
| S2 | H7LH3CCXX_6 | 150711 | FR05812606 | X | 150901 | A | Internal |
| S3 | H7LH3CCXX_7 | 150711 | FR05812622 | X | 150901 | A | Internal |
| S4 | H7LH3CCXX_8 | 150711 | FR05812670 | X | 150901 | A | Edge |
| S5 | H7KMTCCXX_6 | 150711 | FR05812614 | X | 150901 | B | Internal |
| S6 | H7KMTCCXX_7 | 150711 | FR05812662 | X | 150901 | B | Internal |
| S7 | HHKWTCCXX_6 | 151023 | A3 | Y | 151224 | B | Internal |
| S8 | HHKWTCCXX_5 | 151105 | B2 | Y | 151224 | B | Internal |
| S9 | HHKWTCCXX_7 | 151105 | H2 | Y | 151224 | B | Internal |

**Table S13 Concordance of microarray calls to WGS**

The concordance of CNV calls made by aCGH compared to *ClinSV* using WGS. Concordant calls were subset to those that were accurate, imprecise, or disrupted by a diploid region found by WGS. The aCGH extra calls were those identified only in the aCGH data, and these were split into whether they were a common CNV, not reproduced on dye swap, less then 4 aCGH probes, poor deviation or noisy, and being shorter then 1kb. Some discordant CNVs fall in multiple categories but were only listed in one; for example, many common CNV also had poor deviation or low number of probes.

| CNV group | | Number (% of total) | | |
| --- | --- | --- | --- | --- |
|  |  | Agilent 60K CGH  Cohort A | Agilent 400k CGH  Cohort B |  |
| **Concordant** | | 40 (62%) | 214 (75%) |  |
| Accurate (>50% overlap) | 15 (23%) | | 144 (51%) |  |
| Imprecise (0–50% overlap) | 12 (18%) | | 38 (13%) |  |
| Split in WGS | | 13 (20%) | 32 (11%) |  |
| **Discordant: aCGH extra** | | 25 (38%) | 70 (25%) |  |
| Common CNV | | 4 (6%) | 64 (23%) |  |
| Not reproduced on dye swap | | 4 (6%) | - |  |
| ≤ 4 aCGH probes | | 13 (20%) | 3 (1%) |  |
| Poor deviation or noisy | | 3 (5%) | 3 (1%) |  |
| Short (<1 kb) | | 1 (2%) | 0 (0%) |  |
| **Total** | | 65 (100%) | 284 (100%) |  |
| **Per patient avg.** | | **6** | **18** |  |

**Table S14 Clinically reported CNVs detected using *ClinSV***

*ClinSV* was applied in a clinical setting to 485 patients. This table shows 23 reported CNVs.

| **Variant**  **ID** | **CNV detected** | **Size [kb]** | **Genes involved** | **Classification** |
| --- | --- | --- | --- | --- |
| 1 | chr7(GRCh37):  g.128493831_128499722del | 5.89 | FLNC | Pathogenic |
| 2 | chrX(GRCh37):  g.41559320_41615649del | 56.33 | CASK | Pathogenic |
| 3 | chr15(GRCh37):  g.43888601_43897900del | 9.30 | STRC | Pathogenic |
| 4 | chr4(GRCh37):  g.88984923_88989953del | 5.03 | PKD2 | Pathogenic |
| 5 | chr13(GRCh37):  g.103495659_103498818del | 3.16 | exon 1 of ERCC5 | Pathogenic |
| 6 | chr12(GRCh37):  g.116401108_116405464dup | 4.36 | MED13L | Pathogenic |
| 7 | chr17(GRCh37):  g.34807801_36284200del | 1476.40 | HNF1B (+ others) | Pathogenic |
| 8 | chr17(GRCh37):  g.34807701_36284000del | 1476.30 | HNF1B (+ others) | Pathogenic |
| 9 | chrX(GRCh37):  g.154493359_154493852 | 0.49 | exon 1 of RAB39B | Pathogenic |
| 10 | chr17(GRCh37):  g.34811633_36256357del | 1444.72 | HNF1B (+ others) | Pathogenic |
| 11 | chrX(GRCh37):  g.64847028_64897076del | 50.05 | MSN | Likely Path |
| 12 | chr10(GRCH37):  g.55774658_55874816del | 100.16 | PCDH15 | Likely Path |
| 13 | chr6(GRCh37):  g.152519278_152815821del | 296.54 | SYNE1 | Likely Path |
| 14 | chr16(GRCh37):  g.29560701_30199900del | 639.20 | Several | Likely Path |
| 15 | chr15(GRCh37):  g.22748251_23301850del | 553.60 | TUBGCP5, CYFIP1,  NIPA2, NIPA1 | Known susceptibility  variant |
| 16 | chr6(GRCh37):  g.118591129_119144015dup | 552.89 | PLN | VUS |
| 17 | chrX(GRCh37):  g.153296006_153379423dup | 83.42 | MECP2 | VUS |
| 18 | chr19(GRCh37):  g.54300454_54302394dup | 1.94 | NLRP12 | VUS |
| 19 | chr19(GRCh37):  g.50,925120_51030720dup | 105.60 | SPIB | VUS |
| 20 | chr6(GRCh37):  g.76617972_76862874del | 244.90 | MYO6 | VUS |
| 21 | chr16(GRCh37):  g.89595397_89596343del | 0.95 | SPG7 | Pathogenic |
| 22 | chr6(GRCh37):  g.56644527_56806523del | 162.00 | DST | Pathogenic |
| 23 | chr10(GRCh37):  g.68151549_68356392del | 204.84 | CTNNA3 | VUS |

**Table S15 Callability of clinically reported variants by microarrays**

The microarray callability was assessed using the probe location and cut-offs for 5 clinical microarrays currently employed in Australia. 15/23 reported variants were called by some and of these only 10/23 by all clinical microarrays.

| Variant  ID | Size  [kb] | Affy-  metrix CytoScan 750k | Agilent G3 ISCA v3 4x180k | Illumina CytoSNP-  12 | Illumina CytoSNP-  850K | Illumina Infinium GSA v2 | Callable |
| --- | --- | --- | --- | --- | --- | --- | --- |
| 1 | 5.89 | 1 | 0 | 0 | 5 | 3 | N |
| 2 | 56.33 | 52 | 13 | 8 | 21 | 9 | P |
| 3 | 9.30 | 16 | 1 | 0 | 18 | 5 | P |
| 4 | 5.03 | 11 | 2 | 0 | 4 | 4 | N |
| 5 | 3.16 | 3 | 0 | 1 | 3 | 1 | N |
| 6 | 4.36 | 1 | 0 | 1 | 2 | 0 | N |
| 7 | 1476.40 | 561 | 99 | 247 | 552 | 337 | Y |
| 8 | 1476.30 | 561 | 99 | 247 | 552 | 337 | Y |
| 9 | 0.49 | 0 | 0 | 0 | 0 | 0 | N |
| 10 | 1444.72 | 560 | 99 | 247 | 552 | 337 | Y |
| 11 | 50.05 | 14 | 2 | 0 | 3 | 2 | N |
| 12 | 100.16 | 24 | 11 | 10 | 51 | 35 | P |
| 13 | 296.54 | 73 | 18 | 34 | 149 | 202 | Y |
| 14 | 639.20 | 167 | 27 | 101 | 252 | 98 | Y |
| 15 | 553.60 | 297 | 85 | 138 | 150 | 97 | Y |
| 16 | 552.89 | 146 | 27 | 30 | 112 | 104 | Y |
| 17 | 83.42 | 114 | 25 | 17 | 146 | 200 | P |
| 18 | 1.94 | 1 | 0 | 0 | 0 | 2 | N |
| 19 | 105.60 | 26 | 4 | 6 | 46 | 25 | P |
| 20 | 244.90 | 64 | 9 | 22 | 47 | 51 | Y |
| 21 | 0.95 | 0 | 0 | 1 | 1 | 0 | N |
| 22 | 161.00 | 55 | 8 | 24 | 44 | 38 | Y |
| 23 | 204.84 | 29 | 15 | 39 | 64 | 50 | Y |

**Supplemental Figures**


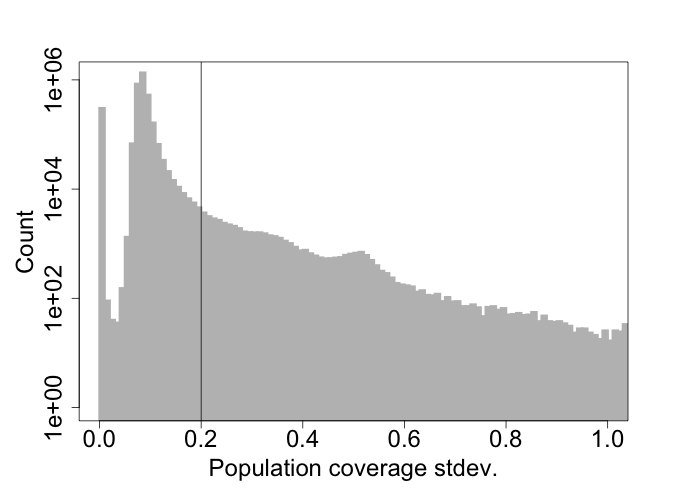


**Fig. S1 Histogram of population coverage standard deviation**

The variability of read depth coverage from 500 controls is expressed as the standard deviation in 1kb windows. Regions with a standard deviation greater than 0.2 were considered frequently deleted or duplicated, representing 2% of the human genome.


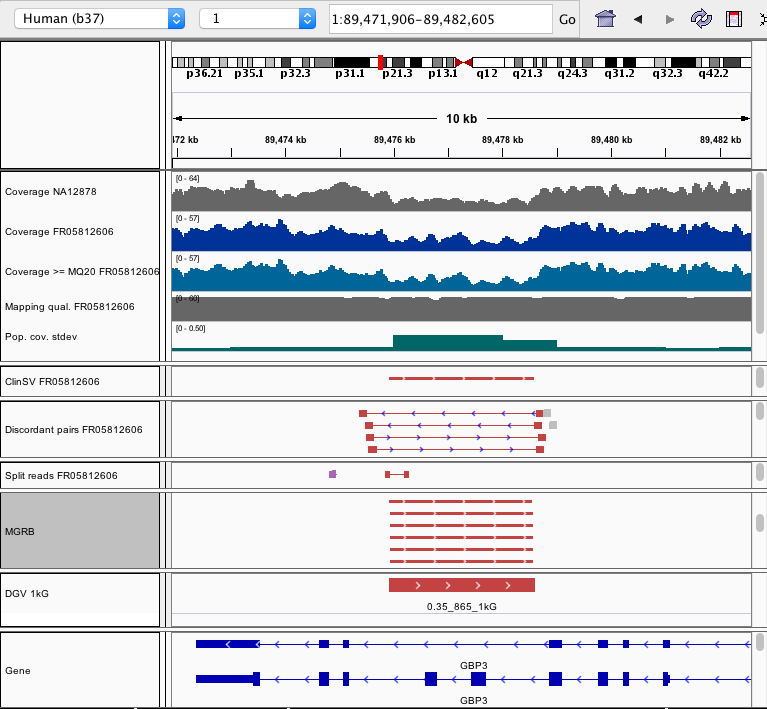


**Fig. S2 Population allele frequency estimates from variant calls**

After sequencing 500 healthy controls with WGS (MGRB) and applying *ClinSV*, we calculated four population allele frequencies (PAFs) from the *ClinSV* variant calls. PAFV is the frequency of *ClinSV* variants called in the population, here 0.62 (track MGRB). PAF1KG is the frequency of CNVs called in the 1000 Genomes Project, here 0.35 (track DGV 1kG). Not shown in this figure are the PAFSU, here 0.71, and PAFDRA, here 0.55, which are derived from the raw split and spanning reads and DOC respectively (see methods).


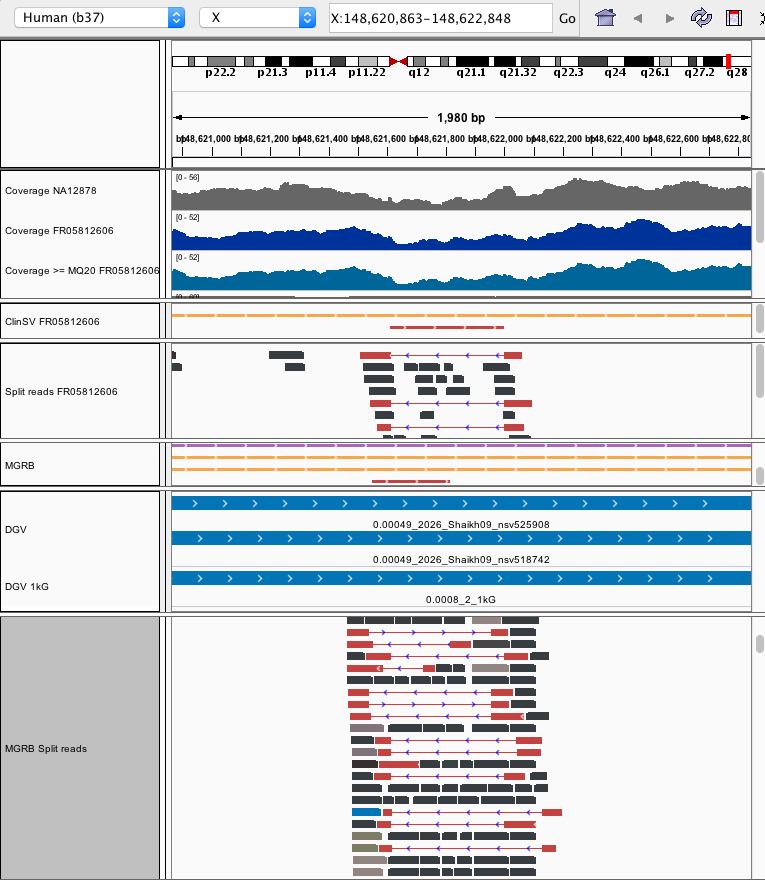


**Fig. S3 Benefit of PAFSU, based on raw SR and DP reads in controls**

*ClinSV* identified a 387 bp deletion (*ClinSV* track), with DOC and SR support in a patient, which would be classified as rare if only the PAF based on final MGRB variant calls are considered, as the PAFV=0.0. However, examination of the split reads in the MGRB cohort (track MGRB split reads) shows that this is a far more frequent event, with a PAFSU of 0.33. The incorporation of raw read evidence from healthy controls allows false positives, or common variants to be correctly excluded.


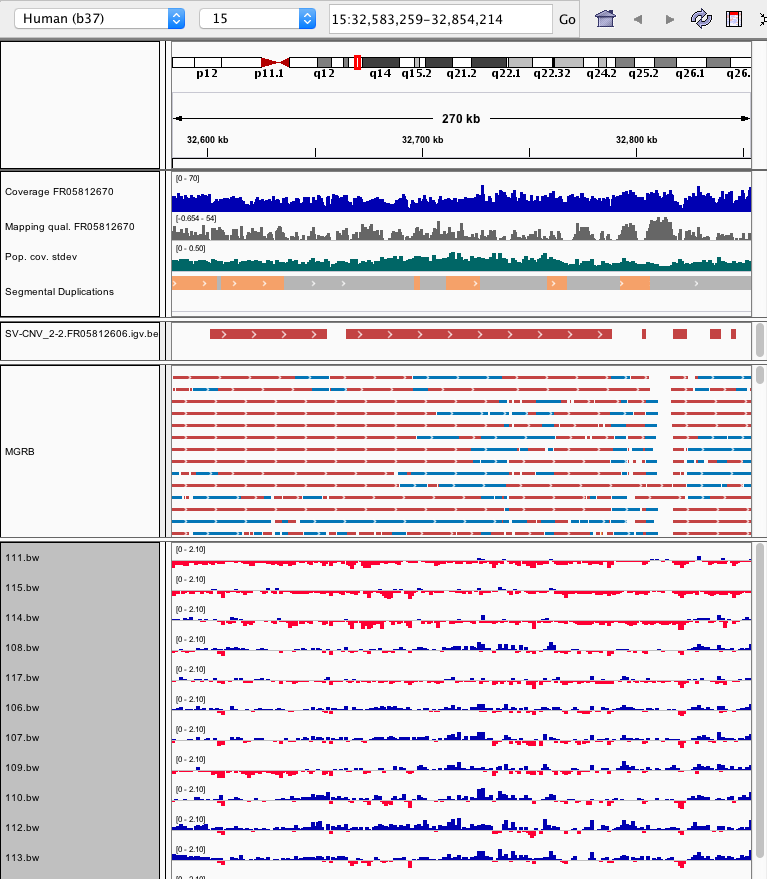


**Fig. S4 Benefit of PAFDRA, based on DOC changes in controls**

*ClinSV* identified a 123.8 kb deletion, with minimal DOC support, which would be classified as rare, if only the final MGRB variant calls are considered, i.e. with a PAFV of 0.002. However, this is a region with poor mapping quality, leading to variable coverage in the patient, and the control population (‘pop cov. stdev' track). Furthermore, many different CNV with different start and end coordinates are found in the MGRB cohort (track MGRB), indicating that the actual PAF is likely higher than estimated by PAFV. PAFDRA is calculated from the normalized average read depth ratio (DRA) of the MGRB cohort in 1kb windows and is 0.08 for this variant, capturing that this is a common CNV, or a false positive. The bottom tracks 106.bw to 117.bw show the DRA for 11 randomly selected MGRB control samples, ordered to show samples with a decreased copy number on top.


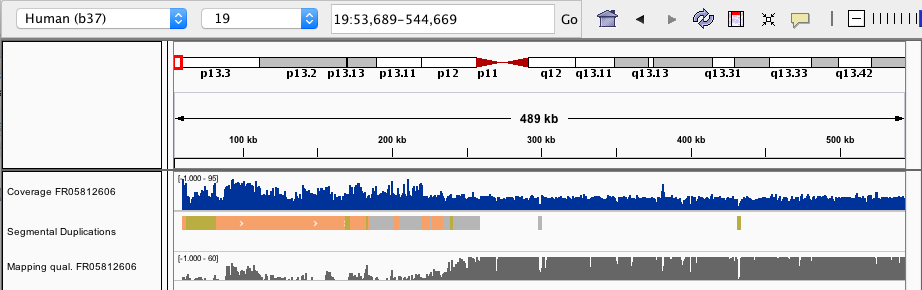


**Fig. S5**

**Depth of coverage variability in regions of segmental duplications**

In a region of known segmental duplication, the coverage becomes spiky, whilst the mapping quality drops.


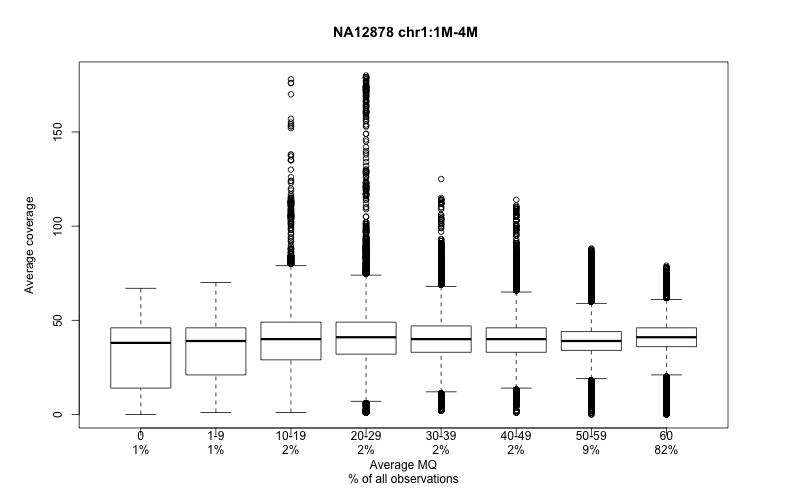


**Fig. S6 Average coverage vs. mapping quality (MQ)**

A boxplot of the average depth of coverage (y-axis) across a 3MB window on chromosome 1 stratified by mapping quality tranches (x-axis), and also indicating the fraction of the 3MB window that falls in each tranche (lower x-axis).


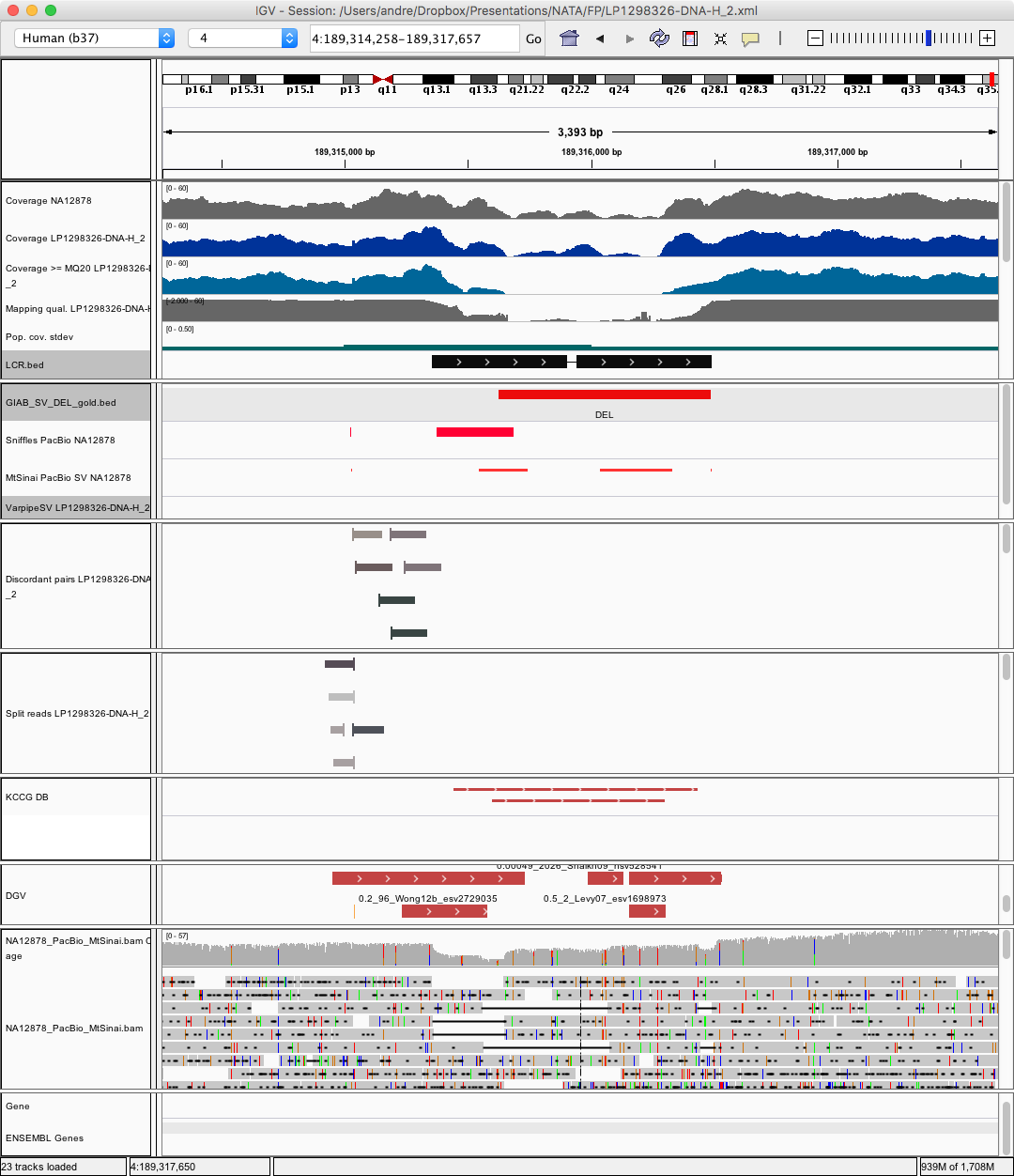


**Fig. S7 GIAB deletion call in repeat region missed by *ClinSV***

An IGV screenshot showing GIAB deletion at chr4:189,315,627-189,316,487. A pair of repeated sequences at the breakpoint are depicted in the LCR.bed track. No DP nor SR supporting the deletion were detected, which are required to detect CNV < 10kb. Raw long-read PacBio data are shown in the bottom tracks.


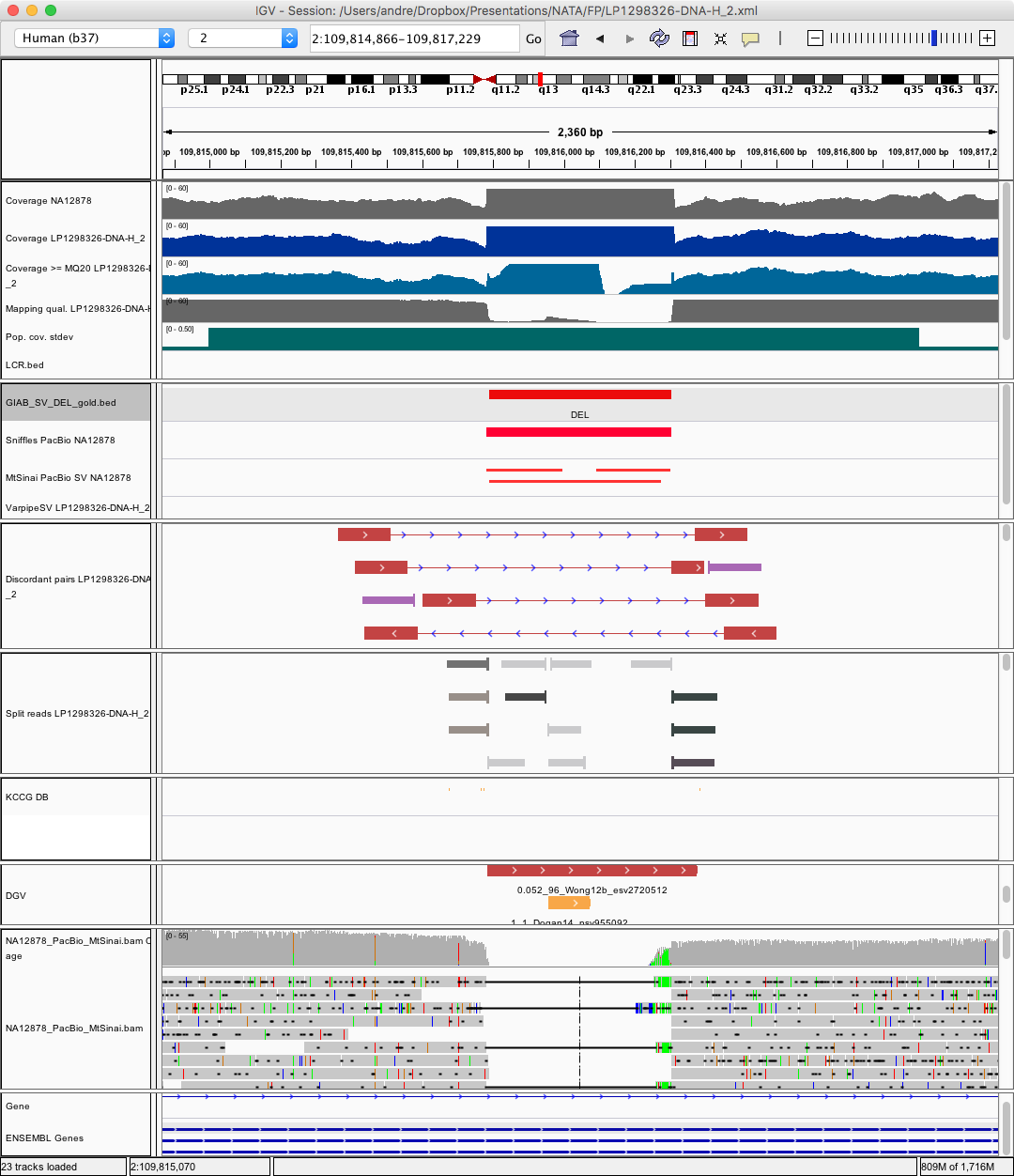


**Fig. S8 GIAB deletion missed by *ClinSV***

*ClinSV* failed to automatically identify a deletion at chr2:109,815,790-109,816,305, which was reported by GIAB (track 7, red), and supported by PacBio data (track 8-9, red; bottom coverage and reads tracks, grey). The short-read sequencing data shows conflicting evidence: the discordant reads indicate a deletion, the split reads indicate an insertion, which maps to different chromosomes (different shades of grey), whereas the DOC increases sharply, indicating a copy number gain. The DOC increase is likely due to misaligned reads aligning to the repeat that has been deleted in this region, but that exists at high copy number in other parts of the genome. The low mapping quality, high coverage in a control, and high population coverage variability (pop. cov. stdev) indicates that this CNV is wholly contained within a repetitive region.


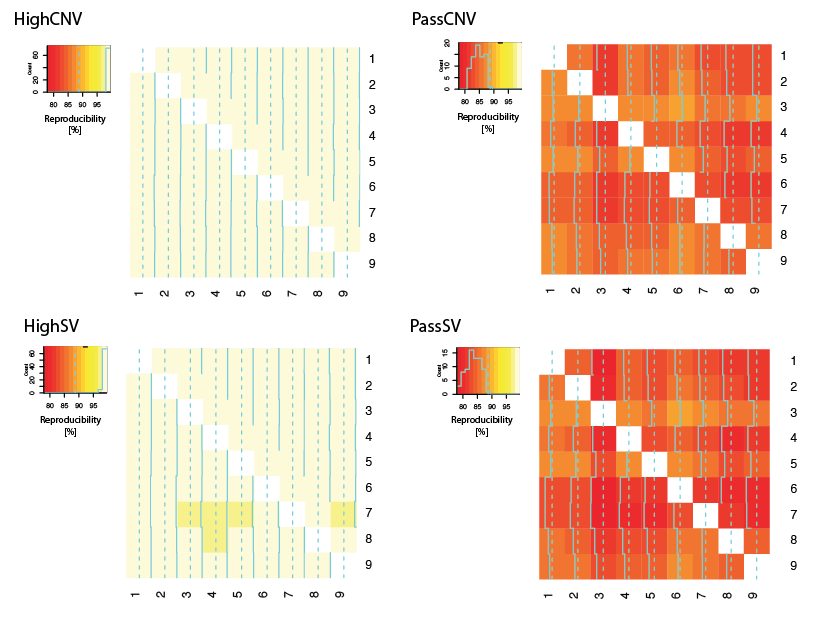


**Fig S9 Reproducibility of *ClinSV***

Nine replicates of NA12878 were sequenced, *ClinSV* was run, and the pairwise concordance of the High or Pass quality CNVs or SVs (including CNVs) variants identified from each run are plotted as a heatmap. See Table S12 for a summary of these concordance values.


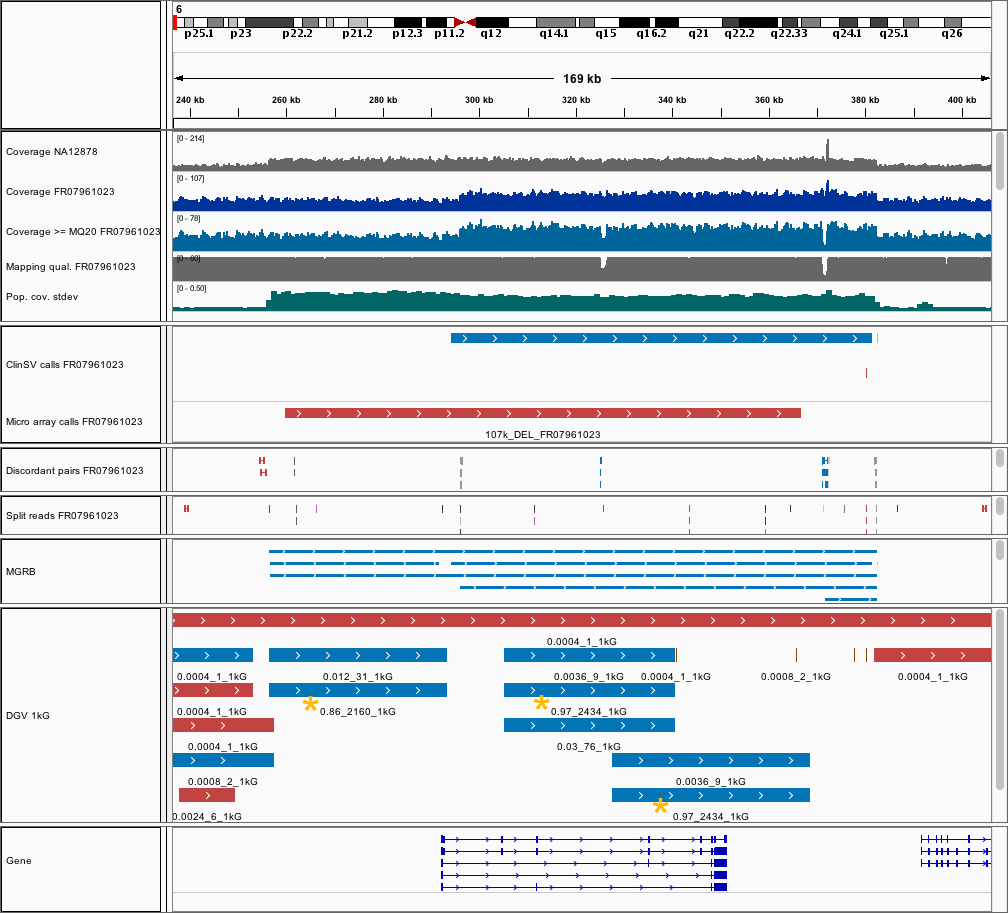


**Fig. S10 False positive microarray deletion in a frequently duplicated region**

The aCGH (track ‘Micro array calls’) identified a deletion, whereas *ClinSV* identifies a duplication affecting a different but largely overlapping region (see the ‘*ClinSV* calls FR07961023’ and ‘Coverage FR07961023’ tracks). The ‘MGRB’, ‘Pop. cov stdev.’ and ‘DGV 1kG’ track provide evidence that region chr6:255kb-295kb is frequently duplicated in the population. Three duplications reported by 1000 Genomes covering most of this region had a population frequency >0.86 (segments with orange stars in the ‘DGV 1kG’ track).


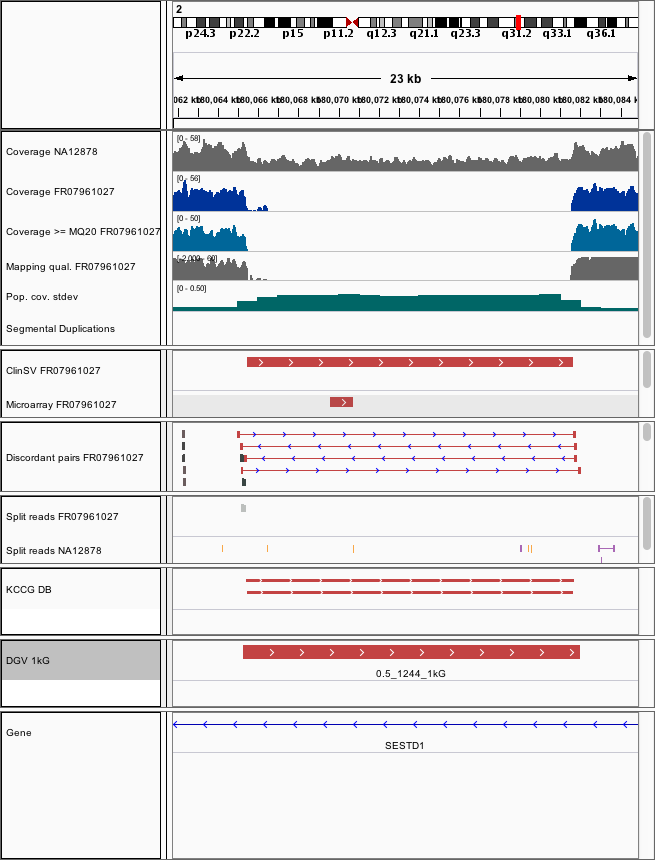


**Fig. S11 An imprecise microarray deletion**

The aCGH microarray detected a much smaller deletion than *ClinSV* using WGS, likely due to the far lower probe density on the microarray in this region compared to WGS.


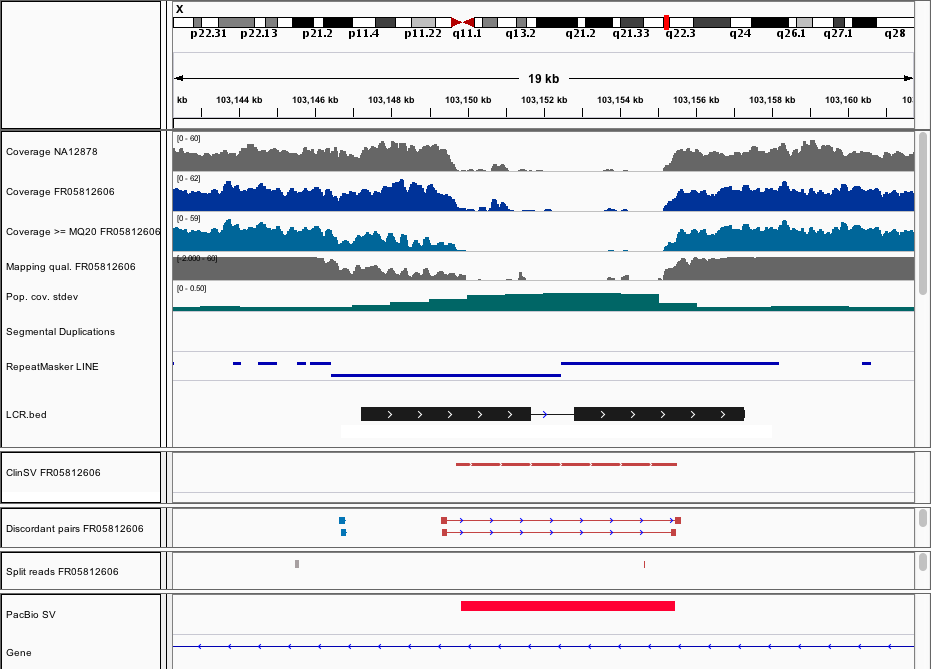


**Fig. S12 Reduced evidence due to repeats at breakpoints**

*ClinSV* identified a deletion CNV (chrX:103,149,701-103,155,500), with two supporting discordant read pairs and no split reads. The mapping quality drop indicates that this is likely a repetitive region, which according to RepeatMasker contains two successive LINE elements (track ‘RepeatMasker LINE’). Aligning the region against itself revealed large parts of the LINE element to show high sequence similarity (95%, track ‘LCR.bed’). The deletion was confirmed with PacBio data (track PacBio SV, see methods).


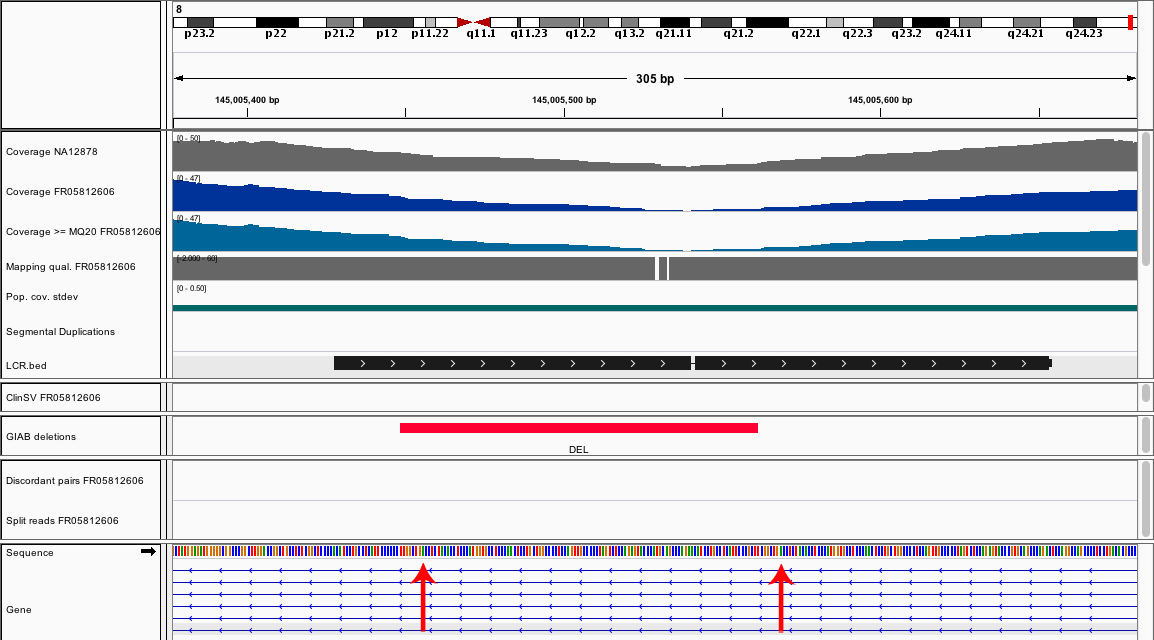


**Fig. S13 GIAB deletion call in tandem repeat region missed by *ClinSV***

The GIAB gold standard contains a deletion (red segment in ‘GIAB deletions’ track) in this tandem repeat region. The LCR.bed track shows that the CNV is surrounded by two copies of the same tandem repeat (see methods), which can also be identified by eye in the sequence track, highlighted by red arrows. Whilst the coverage did decrease in the WGS data, *ClinSV* did not call the variant, as the CNV is <10 kb and there are no supporting DP nor SR were present. The absence of supporting DP/SR reads can be explained by short-read mapping algorithms favoring un-gapped over gapped alignments.


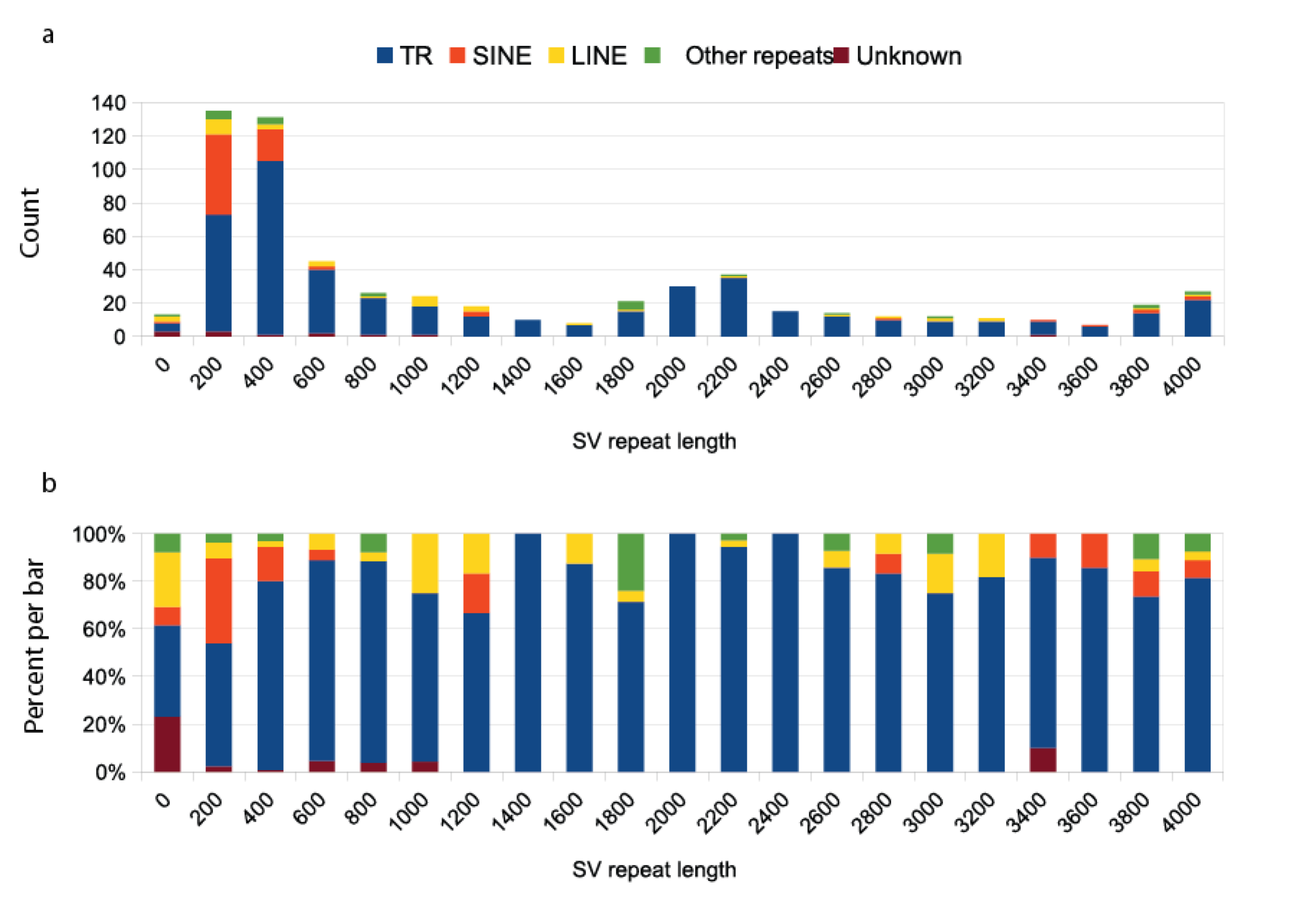


**Fig. S14 Repeat length distribution of repeats surrounding breakpoints**

Histogram showing the lengths of n=625 repeat-pairs surrounding SV breakpoints (a; see methods). SV repeat lengths from 100–700 bp account for 50% of the repeats. The percentage of each class of repeat is shown in panel (b). TR: tandem repeat, SINE: short interspersed nuclear elements, LINE: long interspersed nuclear elements.


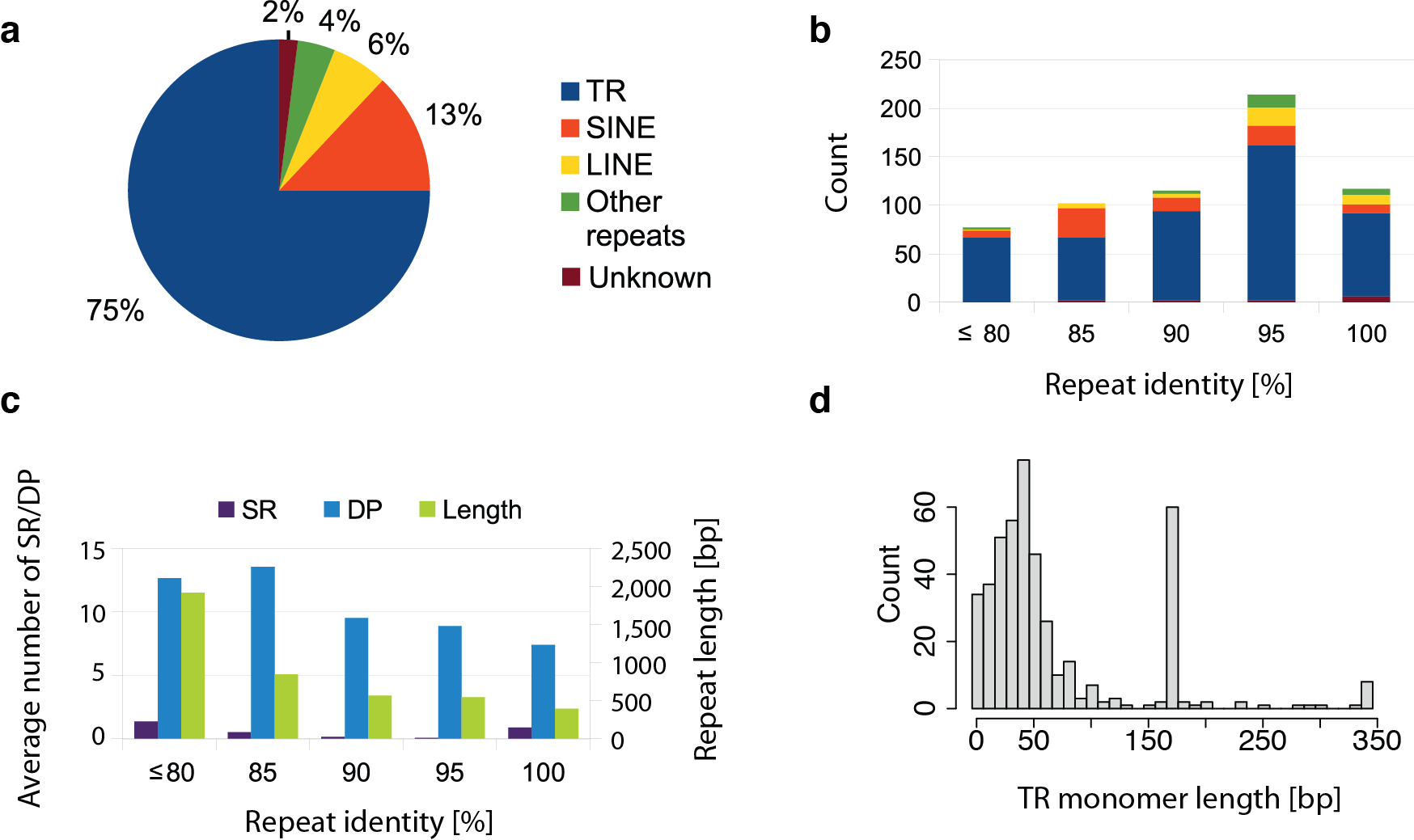


**Fig. S15 CNV with surrounding repeats**

We aligned pairs of genomic sequences surrounding CNV breakpoints from n=4,634 CNV to each other and identified 625 repeat-pairs (see methods). We classified the type of each repeat-pair (**a**), assessed the sequence identity of each repeat-pair (**b**), the average number of split reads (SR) and discordant pairs (DP) and length relative to repeat identity (**c**), and examined the tandem repeat monomer length (**d**). TR: tandem repeat, SINE: Short interspersed nuclear elements, LINE: long interspersed nuclear elements.
