# Supplementary material for: ClinSV: clinical grade structural and copy number variant detection from whole genome sequencing data
Source: Genome Med. 2021 Feb 25;13:32. doi: 10.1186/s13073-021-00841-x (PMC7908648; doi:10.1186/s13073-021-00841-x)
Supplement: Supplementary file 3 — Additional file 3. Sample automated QC report NA12878. ClinSV’s automated QC report for control sample NA12878. [file 13073_2021_841_MOESM3_ESM.pdf]

SV evaluation report sample FR05812606

March 17, 2020

μ mean in control, σ stdev in control, z z-score. OK if $|z| \leq 2$, else !!!

1 QC from bam alignment file

Input bam file: FR05812606.bam
Percent reads pairs not mapping as proper pair: 1.3 [μ 2.1, σ 1.5, z -0.5] OK
Percent reads pairs mapping on different chromosomes: 0.68 [μ 1.2, σ 1, z -0.5] OK
Stdev of coverage: 8.1 [μ 8.1, σ 0.6, z 0] OK

Mapping distance distribution:

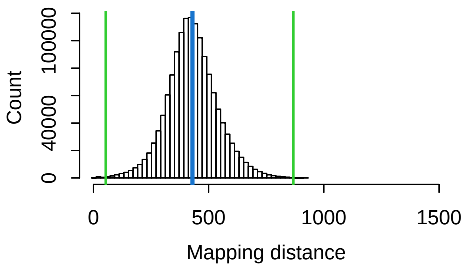

Concordant size min (green): 54 [μ 54, σ 6.7, z 0] OK
Concordant size max (green): 867 [μ 895, σ 60, z -0.5] OK
Mean mapping distance size (blue): 430 [μ 444, σ 26, z -0.5] OK
Stdev mapping distance size: 110 [μ 109, σ 7.4, z 0.1] OK

2 Input discordant pairs and split reads

Input discordant pairs: 10181760 [μ 12025111, σ 7866150, z -0.2] OK
Input split reads: 1032913 [μ 1122920, σ 174515, z -0.5] OK

3 Coverage by chromosome

Average sequence coverage: autosomes 38.4x, X 38.4x, Y 0x
Inferred gender: XX (XX female, XY male, XXY Klinefelter's Syndrome)

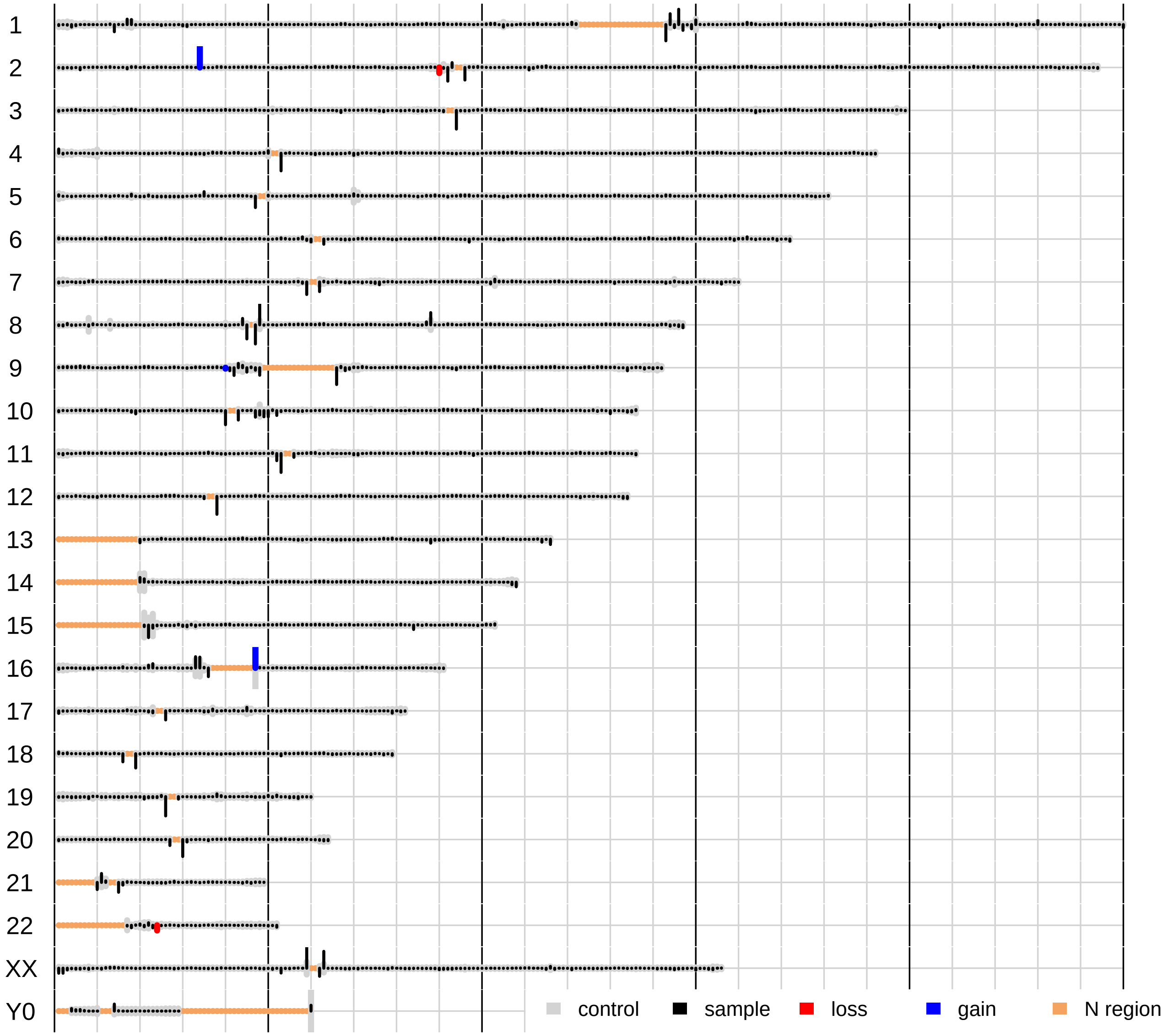

The coverage is normalised by the autosomal average. If the coverage of Y is close to 0, Y0 is plotted representing the background noise. This enables to detect a partial presence of Y.

4 Number called SVs

By call confidence:

High confidence variant: 2044 [μ 1844, σ 106, z 1.9] OK[b]

Pass and High confidence variants: 5741 [μ 5841, σ 323, z -0.3] OK

Low confidence variants: 4516 [μ 3983, σ 666, z 0.8] OK

By SV type [a]:

CNVs: 4634 [μ 4730, σ 187, z -0.5] OK

Loss: 3959 [μ 4025, σ 173, z -0.4] OK

Gains: 675 [μ 705, σ 54, z -0.6] OK

Balanced events including inversions and translocations: 1107 [μ 1110, σ 147, z 0] OK

By caller [a]:

Lumpy calls: 4171 [μ 3492, σ 255, z 2.7] OK[b]

CNVnator calls: 848 [μ 865, σ 37, z -0.5] OK[b]

In both: 722 [μ 1484, σ 87, z -8.8] !!![b]

Gene affecting / rare [a]:

Affecting genes: 2997 [μ 2832, σ 170, z 1] OK

Rare Pass and High confidence: 86 [μ 41, σ 23, z 2] OK[b]

Rare Pass, High confidence, affecting genes: 51 [μ 22, σ 12, z 2.4] OK[b]

Rare Pass, High confidence CNV, affecting genes: 41 [μ 17, σ 8, z 3] OK[b]

[a] Without low confidence variants

[b] OK if $|z| \leq 4$

5 Sensitivity

Compared to NA12878 NIST gold standard consisting of 2664 deletions.
OK if $z \leq$ -2

Table 1: Concordance to 2664 DEL calls from GIAB NA12878

Size	Gold standard calls	Concordant calls	Expected
0-500	1854	1584 (85.4 %)	μ 1560.2, σ 32.6, z 0.73 OK
500-1k	209	203 (97.1 %)	μ 202.7, σ 1.3, z 0.23 OK
1k-5k	435	427 (98.2 %)	μ 429.2, σ 1.9, z -1.2 OK
5k-10k	131	129 (98.5 %)	μ 129.3, σ 0.9, z -0.33 OK
10k-50k	33	33 (100 %)	μ 32.8, σ 0.4, z 0.5 OK
50k-100k	1	1 (100 %)	μ 1, σ 0, z 0 OK
100k-500k	1	1 (100 %)	μ 1, σ 0, z 0 OK
Total	2664	2378 (89.3 %)	μ 2356.2, σ 34.1, z 0.64 OK

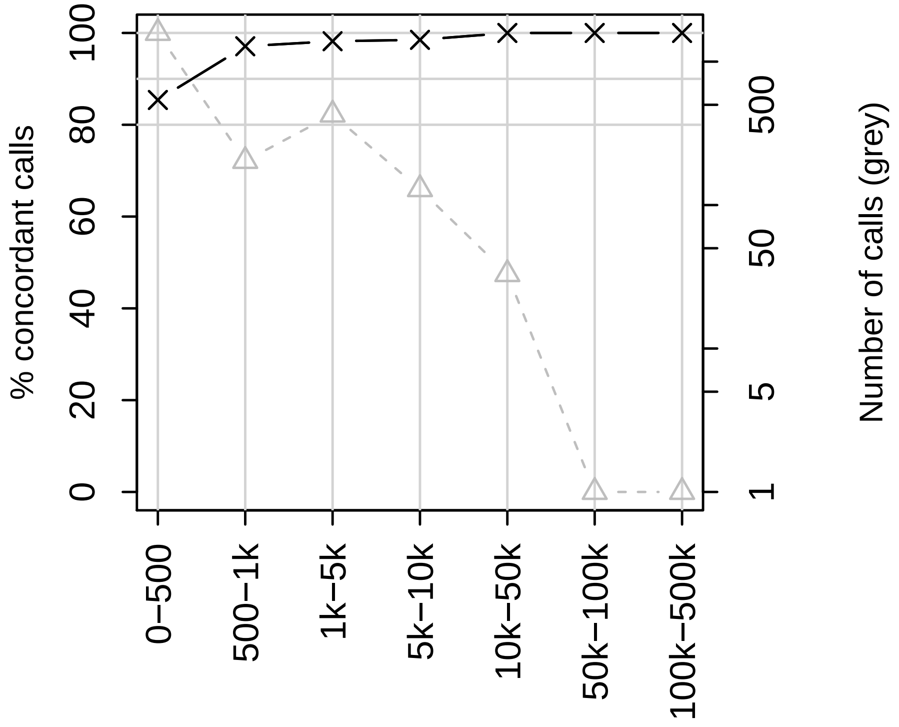

6 Comparison to NA12878 sample

Concordant calls between NA12878 control sample FR05812662 and current sample FR05812606.
Expected values are derived from a set of 8 additional NA12878 control samples. OK if $z \leq$ -2

6.1 Concordance of high confidence variants of control FR05812662

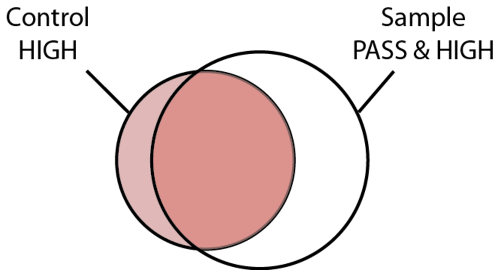

CNV

Size	FR05812662 calls	Concordant calls	Expected
0-500	904	895 (99%)	μ 897.2, σ 2.3, z -0.96 OK
500-1k	175	174 (99.4%)	μ 174.5, σ 0.8, z -0.62 OK
1k-5k	355	353 (99.4%)	μ 351.8, σ 1.8, z 0.67 OK
5k-10k	127	123 (96.9%)	μ 124.6, σ 1.5, z -1.1 OK
10k-50k	77	76 (98.7%)	μ 75.5, σ 0.5, z 1 OK
50k-100k	14	14 (100%)	μ 14, σ 0, z 0 OK
100k-500k	64	61 (95.3%)	μ 61.2, σ 1.7, z -0.12 OK
500k-5M	4	3 (75%)	μ 3.2, σ 0.7, z -0.29 OK
5M-100M	0	0 (NaN%)	μ 0, σ 0, z 0 OK
Total	1720	1699 (98.8%)	μ 1702.1, σ 5, z -0.62 OK

SV

Size	FR05812662 calls	Concordant calls	Expected
0-500	914	904 (98.9%)	μ 906, σ 2.3, z -0.87 OK
500-1k	194	193 (99.5%)	μ 192.6, σ 1.1, z 0.36 OK
1k-5k	417	414 (99.3%)	μ 413, σ 1.9, z 0.53 OK
5k-10k	134	130 (97%)	μ 131.6, σ 1.5, z -1.1 OK
10k-50k	85	84 (98.8%)	μ 83.4, σ 0.5, z 1.2 OK
50k-100k	15	15 (100%)	μ 15, σ 0, z 0 OK
100k-500k	66	63 (95.5%)	μ 63.2, σ 1.7, z -0.12 OK
500k-5M	11	10 (90.9%)	μ 9.9, σ 0.8, z 0.12 OK
5M-100M	55	53 (96.4%)	μ 53.6, σ 0.9, z -0.67 OK
Total	1898	1873 (98.7%)	μ 1875.2, σ 5.6, z -0.39 OK

6.2 Concordance of high confidence and pass variants of control FR05812662

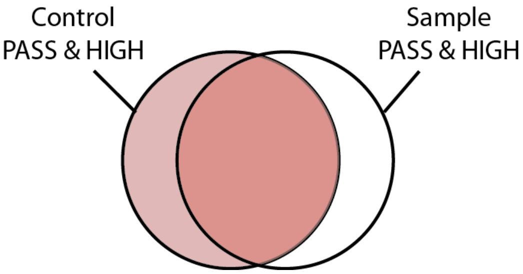

CNV

Size	FR05812662 calls	Concordant calls	Expected
0-500	2270	1983 (87.4%)	μ 1981, σ 43.5, z 0.046 OK
500-1k	506	421 (83.2%)	μ 426, σ 9.1, z -0.55 OK
1k-5k	671	633 (94.3%)	μ 627.6, σ 10.8, z 0.5 OK
5k-10k	198	185 (93.4%)	μ 184.9, σ 2.2, z 0.045 OK
10k-50k	606	448 (73.9%)	μ 444, σ 17, z 0.24 OK
50k-100k	131	121 (92.4%)	μ 116.8, σ 2.4, z 1.8 OK
100k-500k	86	82 (95.3%)	μ 80.8, σ 1.7, z 0.71 OK
500k-5M	13	9 (69.2%)	μ 10.4, σ 1.7, z -0.82 OK
5M-100M	1	1 (100%)	μ 0.9, σ 0.4, z 0.25 OK
Total	4482	3883 (86.6%)	μ 3872.2, σ 66, z 0.16 OK

SV

Size	FR05812662 calls	Concordant calls	Expected
0-500	2300	2007 (87.3%)	μ 2002.1, σ 44.4, z 0.11 OK
500-1k	541	455 (84.1%)	μ 457.9, σ 9.7, z -0.3 OK
1k-5k	794	740 (93.2%)	μ 738.1, σ 14.4, z 0.13 OK
5k-10k	237	220 (92.8%)	μ 220.4, σ 3.6, z -0.11 OK
10k-50k	701	534 (76.2%)	μ 525.9, σ 19.9, z 0.41 OK
50k-100k	214	196 (91.6%)	μ 192.5, σ 2.5, z 1.4 OK
100k-500k	191	174 (91.1%)	μ 170.1, σ 6.5, z 0.6 OK
500k-5M	142	118 (83.1%)	μ 119.1, σ 6.6, z -0.17 OK
5M-100M	288	225 (78.1%)	μ 232.1, σ 11.8, z -0.6 OK
Total	5456	4707 (86.3%)	μ 4695.9, σ 96.2, z 0.12 OK

6.3 Concordance of high confidence variants of test sample FR05812606

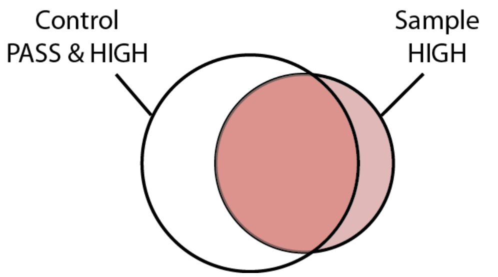

CNV

Size	FR05812606 calls	Concordant calls	Expected
0-500	1034	1022 (98.8%)	μ 982.5, σ 67.9, z 0.58 OK
500-1k	174	174 (100%)	μ 177.4, σ 5.1, z -0.67 OK
1k-5k	362	357 (98.6%)	μ 356.6, σ 6.2, z 0.065 OK
5k-10k	127	125 (98.4%)	μ 127.5, σ 3.4, z -0.74 OK
10k-50k	82	80 (97.6%)	μ 81.8, σ 3.9, z -0.46 OK
50k-100k	12	12 (100%)	μ 13, σ 1.5, z -0.67 OK
100k-500k	61	59 (96.7%)	μ 59.1, σ 2.9, z -0.034 OK
500k-5M	3	3 (100%)	μ 2.8, σ 0.9, z 0.22 OK
5M-100M	0	0 (NaN%)	μ 0, σ 0, z 0 OK
Total	1855	1832 (98.8%)	μ 1800.6, σ 71.9, z 0.44 OK

SV

Size	FR05812606 calls	Concordant calls	Expected
0-500	1046	1032 (98.7%)	μ 989.9, σ 68.4, z 0.62 OK
500-1k	191	190 (99.5%)	μ 195.4, σ 8.1, z -0.67 OK
1k-5k	426	420 (98.6%)	μ 419.2, σ 7.4, z 0.11 OK
5k-10k	134	132 (98.5%)	μ 136.6, σ 4.6, z -1 OK
10k-50k	92	90 (97.8%)	μ 90.5, σ 5, z -0.1 OK
50k-100k	14	14 (100%)	μ 15, σ 1.5, z -0.67 OK
100k-500k	63	61 (96.8%)	μ 61.1, σ 2.9, z -0.034 OK
500k-5M	11	11 (100%)	μ 9.5, σ 1.4, z 1.1 OK
5M-100M	57	52 (91.2%)	μ 56.5, σ 6.4, z -0.7 OK
Total	2044	2010 (98.3%)	μ 1982.8, σ 79.5, z 0.34 OK

6.4 Concordance of high confidence and pass variants of test sample FR05812606

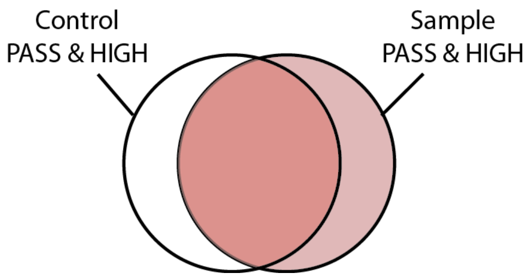

CNV

Size	FR05812606 calls	Concordant calls	Expected
0-500	2414	1989 (82.4%)	μ 1988.5, σ 51.8, z 0.0097 OK
500-1k	498	423 (84.9%)	μ 423.8, σ 17.4, z -0.046 OK
1k-5k	679	627 (92.3%)	μ 625.1, σ 13.1, z 0.15 OK
5k-10k	205	182 (88.8%)	μ 184, σ 3.4, z -0.59 OK
10k-50k	608	485 (79.8%)	μ 490.9, σ 24.6, z -0.24 OK
50k-100k	128	116 (90.6%)	μ 116.9, σ 6.8, z -0.13 OK
100k-500k	89	81 (91%)	μ 79, σ 3.6, z 0.56 OK
500k-5M	11	9 (81.8%)	μ 10.1, σ 1.9, z -0.58 OK
5M-100M	2	1 (50%)	μ 0.9, σ 0.4, z 0.25 OK
Total	4634	3913 (84.4%)	μ 3919.1, σ 70.9, z -0.086 OK

SV

Size	FR05812606 calls	Concordant calls	Expected
0-500	2449	2013 (82.2%)	μ 2007.4, σ 53.4, z 0.1 OK
500-1k	532	454 (85.3%)	μ 455.4, σ 18.3, z -0.077 OK
1k-5k	799	732 (91.6%)	μ 737.6, σ 16.2, z -0.35 OK
5k-10k	245	216 (88.2%)	μ 218.2, σ 4.9, z -0.45 OK
10k-50k	729	579 (79.4%)	μ 574.4, σ 31.3, z 0.15 OK
50k-100k	221	196 (88.7%)	μ 195, σ 8.6, z 0.12 OK
100k-500k	218	180 (82.6%)	μ 171.6, σ 10.1, z 0.83 OK
500k-5M	155	115 (74.2%)	μ 116.2, σ 8.8, z -0.14 OK
5M-100M	340	239 (70.3%)	μ 246.8, σ 21.3, z -0.37 OK
Total	5741	4762 (82.9%)	μ 4760, σ 129.6, z 0.015 OK

7 Explanation QC and SV evaluation report

7.1 General QC

Low quality input data may lead to unexpected results. To guarantee the quality of the results several variables that have an impact on or are an indicator for the quality of the results are measured and compiled in this automated QC report. QC metrics measured for a particular sample are compared to the expected range obtained from analyzing 500 germline controls samples. The control samples represent previously analyzed healthy individuals (MGRB cohort) that passed QC. The expected range is generally defined as two times the standard deviation ($|z| \leq 2$) from the mean of the control cohort, unless specified otherwise. If a measured metric is within expectations, it is marked with a green OK, else with three orange exclamation marks. ClinSV is robust to a few metrics being outside the expected range, but within 4 times the standard deviation.

Re 1. QC from bam alignment file

Read pairs from Illumina paired end sequencing do not always align to the reference with their expected distance (roughly 450 bp, depending fragmentation size and size selection), regardless of the presence of structural variation. The sequencing process produces a small percentage of chimeric read pairs. These pairs originate from distant genomic locations. Despite these chimeric reads being randomly distributed; elevated numbers will impact the SV calling. Indicators for the relative abundance of chimeric pairs is the percentage of reads not mapping as proper pairs and the percentage of pairs mapping on different chromosomes. An uneven read coverage can affect CNVnator resulting in an elevated number of false CNV calls. The un-evenness of the read coverage is reflected by an increased standard deviation of the read coverage. The number of discordantly mapping pairs the prediction program Lumpy can handle is finite. The threshold for when the read mapping distance is considered discordant for pairs mapping with the expected read orientation is automatically determined (see online methods section), and results are shown here. The insert size distribution and resulting thresholds for concordant mapping distances have an impact on the smallest detectable deletions. To save computing time, metrics in this section are estimated for a 10 mega base pair region on chromosome 1 (chr1:20,000,001-30,000,000).

Re 2. Input discordant pairs and split reads

Number of discordant read pairs and split reads used as input for Lumpy. Deviating numbers could indicate library preparation or sequencing issues, deeper coverage, or samples with high numbers of structural variation, as expected for cancer samples.

Re 3. Coverage by chromosome

The average sequence coverage was determined for all chromosomes (see methods). The number of sex chromosomes is inferred from the sequence coverage. Sex chromosome aneuploidy is visible here. This section also displays the chromosome wide coverage in intervals of 1 Mega bases. Grey dots below the black dots represent the average coverage in 500 control samples plus minus two times the standard deviation. The black dots indicate the coverage of the current sample. Truncated alignment files will not cover all grey dots. One Mega base segments greater than five times the standard deviation of the control are colored blue, highlighting regions that have a copy number gain, and segments less than five times the standard deviation are colored in red, highlighting regions of copy number loss. The standard deviation is used, because regions close to the centromere tend to show a greater variation that is still considered normal, thus will not get highlighted in blue or red. Large deletions or duplications, that are likely clinical significant will be visible in this representation. N-regions usually correspond to centromeric or telomeric regions of the chromosome. The sex chromosomes will be compared to the expected coverage of X, XX, Y and/or Y0 (Y-zero), depending the average coverage of X and Y. Y0 (Y-Zero) indicates unspecific background read coverage of the Y chromosome and is helpful to reveal a partial presence of Y.

Re 4. Number of called SVs

Number of called variants by call confidence, SV type, caller, and number of variants affecting genes and being rare. Some metrics in this section show greater variation and are allowed 4 times the standard deviation from the control average. For

instance the number of rare variants could be increased in an individual of a race underrepresented in the control cohort.

7.2 NA12878 SV evaluation

The following two sections are to evaluate the SV recall rate of a NA12878 sample and allow assessing the fitness of the entire SV detection pipeline. Metrics are compared to average values of nine NA12878 control samples. Here z values greater or equal to -2 are acceptable, in order to not penalize a greater concordance than expected from the nine NA12878 control samples. This section appears when option -eval is set.

Re 5. Sensitivity
This section shows the sensitivity of detecting gold standard deletion calls, as published by GIAB (Parikh et al. 2016), excluding 12 false positives $>$ 500 bases (Minoche et al. 2017).

Re 6. Comparison to NA12878 sample
Concordance of SV between NA12878 control sample (FR05812662) and current test sample, shown in percent of FR05812662 calls or in percent of test sample calls. High confidence calls generally have a higher reproducibility compared to all pass variants. CNVs and all SVs are tested separately.
